# In Situ Growth CNTs and Commercialization MWCNTs Dual-Reinforced MoS_2_ with Cross-Link Structure for Stable Sodium-Ion Storage

**DOI:** 10.3390/ma19173586

**Published:** 2026-08-24

**Authors:** Xiao Li, Nana Hu, Weina Bi, Shilong Wen, Shufan Feng, Xuesong Zhang, Baogang Zhao, Jiaoxian Yu, Jixun Xie, Jingyun Ma

**Affiliations:** 1 Shandong Provincial Key Laboratory of Chemical Energy Storage and Novel Cell Technology, School of Materials Science and Engineering, Qilu University of Technology (Shandong Academy of Sciences), Jinan 250353, China; 17205411032@163.com (X.L.); biweina1107@163.com (W.B.); 19853105480@163.com (S.W.); fengshufan0810@163.com (S.F.); 17515129271@163.com (X.Z.); zhaobaogang12345@163.com (B.Z.); 2 College of Chemistry and Chemical Engineering, Ocean University of China, Qingdao 266100, China; hunn@stu.ouc.edu.cn; 3 Advanced Materials Institute, Qilu University of Technology (Shandong Academy of Sciences), Jinan 250014, China

**Keywords:** sodium-ion batteries, MoS_2_, CNTs, cycling stability, storage mechanism

## Abstract

It is essential to design electrode structures which simultaneously ensure mechanical strength and facilitate rapid sodium-ion transport to enable practical and large-scale sodium-ion battery (SIB) applications. In this study, we report a novel anode material featuring a cross-linked architecture composed of MoS_2_ reinforced internally by catalytically derived CoS_2_@C-supported carbon nanotubes (CNTs), and externally by commercial multi-walled carbon nanotubes (MWCNTs). This dual-reinforced configuration effectively prevents MoS_2_ layer aggregation, enhances structural integrity, and establishes continuous conductive frameworks for efficient electron transmission. Additionally, it offers ample ion-diffusion pathways and mechanical resilience to buffer volume changes during cycling. Density functional theory (DFT) simulations reveal that the modified MoS_2_ structure exhibits a significantly reduced sodium-ion diffusion barrier, contributing to enhanced charge-discharge kinetics. The CoS_2_@C/CNTs@MoS_2_@MWCNTs electrode achieves remarkable cycling stability, retaining 395 mA h g^−1^ at 1 A g^−1^ for 2000 cycles. In situ X-ray diffraction (XRD) along with kinetic analyses confirm a pseudocapacitance-dominated storage mechanism. Furthermore, full coin-type cells assembled with Na_3_V_2_(PO_4_)_3_ cathodes demonstrate excellent cycling performance, demonstrating the practical potential of this design strategy for advanced SIBs.

## 1. Introduction

Addressing the escalating climate crisis driven by human-induced greenhouse gas emissions necessitates the advancement of next-generation, high-efficiency clean energy storage technologies [1,2,3,4]. Among the existing solutions, lithium-ion batteries (LIBs) dominate the markets for portable electronics and electric vehicles due to their impressive energy density (280~350 Wh kg^−1^). However, their reliance on scarce lithium resources, use of flammable electrolytes, and environmentally intensive mining practices have raised concerns over long-term sustainability. In contrast, sodium-ion batteries (SIBs) are gaining traction as a viable alternative, owing to their similar electrochemical behavior, the abundance and wide geographical availability of sodium, improved safety profile, and resilience at low temperatures [5,6,7,8,9]. Despite these advantages, the expanded ionic dimension of sodium-ion (1.02 Å versus 0.76 Å for lithium ion) results in sluggish solid-state diffusion and induces significant structural swelling upon cycling, which accelerates structural degradation and leads to capacity decay over time [10,11,12]. As a result, the evolution of anode architectures with optimized architectures that is capable of hosting fast as well as reversible sodium-ion insertion/extraction remains a pressing research focus [13,14].

Among various candidates, two-dimensional MoS_2_ stands out for its high theoretical capacity (670 mAh g^−1^), ample interlayer spacing (~0.62 nm), and low-strength van der Waals forces between layers, which collectively support sodium-ion intercalation during sodiation [15,16]. Nonetheless, pristine MoS_2_ faces critical drawbacks including low electrical conductivity, a conversion-type storage mechanism, and pronounced structural fragmentation during repeated cycling [17]. To mitigate these limitations, researchers explored several modification strategies. Nanostructural engineering techniques such as heteroatom doping, vacancy creation, and construction of heterostructures have been shown to improve charge transport, increase active site density, and tailor the electronic structure for enhanced ion kinetics and electrochemical performance [18,19]. Additionally, incorporating MoS_2_ into conductive carbon scaffolds—ranging from porous carbon to carbon nanotubes (CNTs) and graphene—can effectively buffer volume expansion and boost conductivity [20,21,22]. Yet, issues such as MoS_2_ nanosheet aggregation and loss of active material still remain. Spatial confinement of MoS_2_ within interconnected porous carbon networks has proven to be one promising route to enhance both mechanical stability and sodium storage capacity [23,24,25,26].

In recent years, spatial confinement has emerged as an effective strategy for prolonging the cycling life of conversion-type anode materials used in sodium-ion storage systems [27,28]. One promising approach involves the construction of three-dimensional porous carbon matrix frameworks, which restrict the electrochemical conversion reactions between MoS_2_ and sodium ions within confined nanoscale spaces. This spatial limitation not only promotes more controlled reactions but also significantly mitigates structural degradation through enhanced anchoring effects [29,30]. Comparative studies have shown that, unlike bulk structures or single-phase nanomaterials, MoS_2_ integrated into porous carbon networks retains the electrochemical activity characteristic of monolayer nanosheets. At the same time, this structure effectively buffers volume changes through mechanical confinement, thereby enhancing cycling stability [31]. Particularly noteworthy is the bilateral confinement enabled by the cross-linked architecture, which facilitates both electron transport and ion mobility. In this design, the carbon matrix on one side primarily accelerates electron conduction; the central confined region hosts the conversion reactions of MoS_2_, absorbing mechanical strain; while the outer carbon layer inhibits polysulfide dissolution, stabilizes the electrode-electrolyte interface, and provides interconnected channels for fast sodium-ion diffusion [32,33,34].

Building on the strategies discussed above, we devised a spatial confinement approach to synthesize a novel CoS_2_@C/CNTs@MoS_2_@MWCNTs composite through a combination of hydrothermal processing and subsequent calcination. This method yielded a hierarchically organized, micro-flower-like structure uniformly enveloped by a continuous CNTs layer. A key innovation of this architecture lies in its dual-reinforcement design: MoS_2_ is supported internally by CoS_2_@C particles and catalytically grown CNTs, while the external shell comprises commercial multi-walled carbon nanotubes (MWCNTs). Together, these components interconnect to form a robust, cross-linked three-dimensional framework. The bilateral confinement imparted by this configuration results in synergistic functional mechanisms: mechanical stabilization through dual CNT reinforcement, efficient transport of both electrons and ions via multi-channel conductive networks, and favorable interfacial electronic interactions. These features collectively contribute to the exceptional electrochemical performance of the electrode. The unique structure of the CoS_2_@C/CNTs@MoS_2_@MWCNTs electrode, as revealed by first-time in situ XRD analysis, synergistically facilitates internal charge transfer and enhances Na^+^ adsorption, a conclusion supported by density functional theory (DFT) calculations. Consequently, the composite achieves a high reversible capacity of 395 mA h g^−1^ after 2000 cycles at 1 A g^−1^, with a retention rate of 89.9%, demonstrating its promise for next-generation SIB applications and offering a compelling model for the structural engineering of two-dimensional energy materials.

## 2. Methods

### 2.1. Material

The substances Co(NO_3_)_2_·6H_2_O and 2-methylimidazole (C_4_H_6_N_2_/2-MIM) were purchased from Shanghai Macklin Biochemical Technology Co, Ltd. (China) and utilized directly without further purification. Other chemicals, including methanol (99.5% purity), thiourea (99.5% purity), Cetyltrimethylammonium Bromide (CTAB), Dicyandiamide (DCDA) ammonium molybdate tetrahydrate (99.5% purity), MWCNT, were all provided by Sinopharm Chemical Reagent Co, Ltd. (China) and also employed as they were.

### 2.2. Synthesis of Co@C/CNTs

Firstly, 2.49 g of cobalt (II) nitrate hexahydrate (Co(NO_3_)_2_·6H_2_O) was dissolved in 250 mL of methanol under stirring for 30 min. Simultaneously, 3.28 g of 2-methylimidazole (C_4_H_6_N_2_) was separately dissolved in another 250 mL of methanol under stirring for 30 min. The two solutions were then combined into a single beaker and stirred vigorously for 1 h. The resulting mixture was allowed to stand undisturbed for 24 h, yielding a purple precipitate. The supernatant was discarded, and the precipitate was collected. The precipitate was washed twice with methanol and then twice with deionized water, each time by centrifugation at 8000 rpm. The obtained solid was dried in an oven at 60 °C for 24 h to obtain the ZIF-67 precursor.

Subsequently, the as-synthesized ZIF-67 was subjected to calcination under a nitrogen atmosphere in a tube furnace. The temperature was raised to 500 °C at a heating rate of 2 °C/min and held for 3 h. The resulting material and dicyandiamide (mass ratio 1:10) were placed in two separate crucibles, with the crucible containing dicyandiamide positioned upstream in the gas flow. Under a nitrogen atmosphere, the temperature was increased to 600 °C at a rate of 2 °C/min and maintained for 3 h, followed by natural cooling. The final product was denoted as Co@C/CNTs.

### 2.3. Preparation of Pure MoS_2_ and CoS_2_@C/CNTs@MoS_2_

Pure MoS_2_ was synthesized as follows: 2 mg of CTAB, 0.76 g of thiourea, and 0.35 g of ammonium molybdate tetrahydrate were dissolved in 60 mL of deionized water and stirred for 30 min. The mixture was then transferred into a Teflon-lined stainless-steel autoclave and maintained at 200 °C for 20 h. After the reaction, the autoclave was allowed to cool naturally to room temperature. The supernatant was decanted, and the precipitate was collected and dried in an oven to obtain the final MoS_2_ product.

For the CoS_2_@C/CNT@MoS_2_ composite, the same amounts of CTAB, thiourea, and ammonium molybdate tetrahydrate as used above were dissolved in 60 mL of deionized water and stirred for 30 min. Then, 90 mg of Co@C/CNTs were dispersed in the above solution and subjected to the identical hydrothermal treatment and post-reaction workup (200 °C, 20 h, natural cooling, decantation, and oven drying) to yield the final product, denoted as CoS_2_@C/CNT@MoS_2_.

### 2.4. Preparation of CoS_2_@C/CNTs@MoS_2_@MWCNTs

Prior to the synthesis, the MWCNTs were activated to improve their surface properties. Specifically, 2 g of MWCNTs were added into a mixture of concentrated sulfuric and nitric acids (volume ratio 3:1) and ultrasonically treated for 30 min. The mixture was then heated to 80 °C within 15 min and refluxed at 80 °C for 3 h. After the reaction, the product was filtered, washed several times with deionized water, and finally freeze-dried to obtain the activated MWCNTs.

In a standard synthesis route for the CoS_2_@C/CNTs@MoS_2_@MWCNTs hybrid material, 60 mg of activated MWCNTs [35] and the previously prepared CoS_2_@C/CNTs@MoS_2_ composite were dispersed in a 60 mL mixed solvent of N,N-dimethylformamide (DMF) and deionized water (*v*/*v* = 2:1) using ultrasonic treatment over 30 min to achieve a uniform suspension. The homogeneous dispersion was then placed in one 100 mL Teflon-lined stainless-steel autoclave and treated solvothermal over 12 h under 200 °C. At the end of the process, the resulting mixture was retrieved through vacuum filtration, repeatedly rinsed with distilled water to purify the product, and subsequently lyophilized to eliminate solvent traces and enhance dispersion quality. The final material obtained was the CoS_2_@C/CNTs@MoS_2_@MWCNTs hybrid.

### 2.5. Material Characterization

X-ray diffraction (XRD: X-ray diffraction patterns were acquired using Cu Kα radiation) patterns were obtained with a MiniFlex600 diffractometer (SHIMADZU, Japan). Morphological along with microstructural features were examined via field-emission scanning electron microscopy (FE-SEM, Hitachi S-520, JXA-840) and high-resolution transmission electron microscopy (HR-TEM, JEM-2100F). High-resolution transmission electron microscopy (HR-TEM) images were obtained using a JEM-2100F microscope (JEOL, Japan) operated at an accelerating voltage of 200 kV. The microscope was equipped with a Schottky field-emission gun, with a point resolution of 0.23 nm and a lattice resolution of 0.10 nm. Specimens for TEM analysis were prepared using a focused ion beam (FIB) instrument (FEI Helios 5 HX). The chemical states and elemental composition were determined via X-ray photoelectron spectroscopy (XPS, Thermo Scientific NEXSA) using a monochromatic Al Kα X-ray source (hν = 1486.6 eV). All binding energies were calibrated relative to the C 1s peak of adventitious carbon at 284.8 eV. The porosity distribution and specific surface interface of pyrolyzed samples were measured using a Gominiv2380 surface area analyzer. X-ray absorption fine structure (XAFS) spectroscopy was carried out using a laboratory XAFS spectrometer (Deep-Inspectra-X1, Beijing SciStar Technology Co., Ltd.) in transmission mode. The instrument was operated at 20 kV and 40 mA, with an energy range of 7012–7712 eV. A Si (531) spherically bent crystal analyzer (radius of curvature: 500 mm) was employed for Co K-edge measurements. The samples were measured in powder form. Additionally, in situ electrochemical impedance spectroscopy (EIS: impedance spectra were acquired in a three-electrode configuration using a metallic sodium reference electrode, measurements were carried out with a Kejing EQ-3ESTC15 three-electrode cell (Kejing, China)) was conducted using the Solartron Analytical Modulab XM PhotoEchem to track real-time variations in internal resistance during charge-discharge cycles, providing insight into the dynamic evolution of electrochemical performance.

### 2.6. Electrochemical Measurement

CR2032-type coin cells were assembled in an argon-filled glove box, employing sodium metal foil as both the counter and reference electrode. The working electrode was fabricated by thoroughly mixing the active material (e.g., CoS_2_@C/CNTs@MoS_2_@MWCNTs 1.1 mg), Super P carbon black, and polyvinylpyrrolidone (PVP) in a mass ratio of 7:2:1 using N-methyl-2-pyrrolidone (NMP) as the solvent. The resulting slurry was further homogenized via ball milling over 6 h, then uniformly coated onto a copper foil current collector and dried at 70 °C over 122h under vacuum. The electrolyte comprised 1 M NaClO_4_ dissolved in a ternary solvent mixture of ethylene carbonate (EC), diethyl carbonate (DEC), and ethyl methyl carbonate (EMC) in a 1:1:1 volume ratio. For full-cell configurations, Na_3_V_2_(PO_4_)_3_ (NVP) was utilized as the cathode material. Electrochemical performance was evaluated through galvanostatic charge-discharge (GCD) tests via a LAND-CT2001 battery test system. Additional measurements, including cyclic voltammetry (CV) and EIS, were performed via a CHI660D electrochemical workstation.

Operando X-ray diffraction (XRD) measurements were carried out to monitor the structural evolution of the electrode material during sodiation/desodiation. Inside an argon-filled glovebox, the working electrode was placed on a beryllium window (serving as the X-ray transparent window), and an operando cell was assembled following the same procedure as for a half-cell. The cell was then mounted on a powder X-ray diffractometer, and XRD patterns were collected in reflection geometry over a 2θ range of 10–45° with a scan time of approximately 8 min per pattern. Galvanostatic charge–discharge was performed at a constant current density of 100 mA g^−1^ within the voltage window of 0.01–3.0 V, and XRD scans were continuously recorded throughout the first cycle to capture real-time phase transformations.

## 3. Results and Discussion

**Figure 1:**
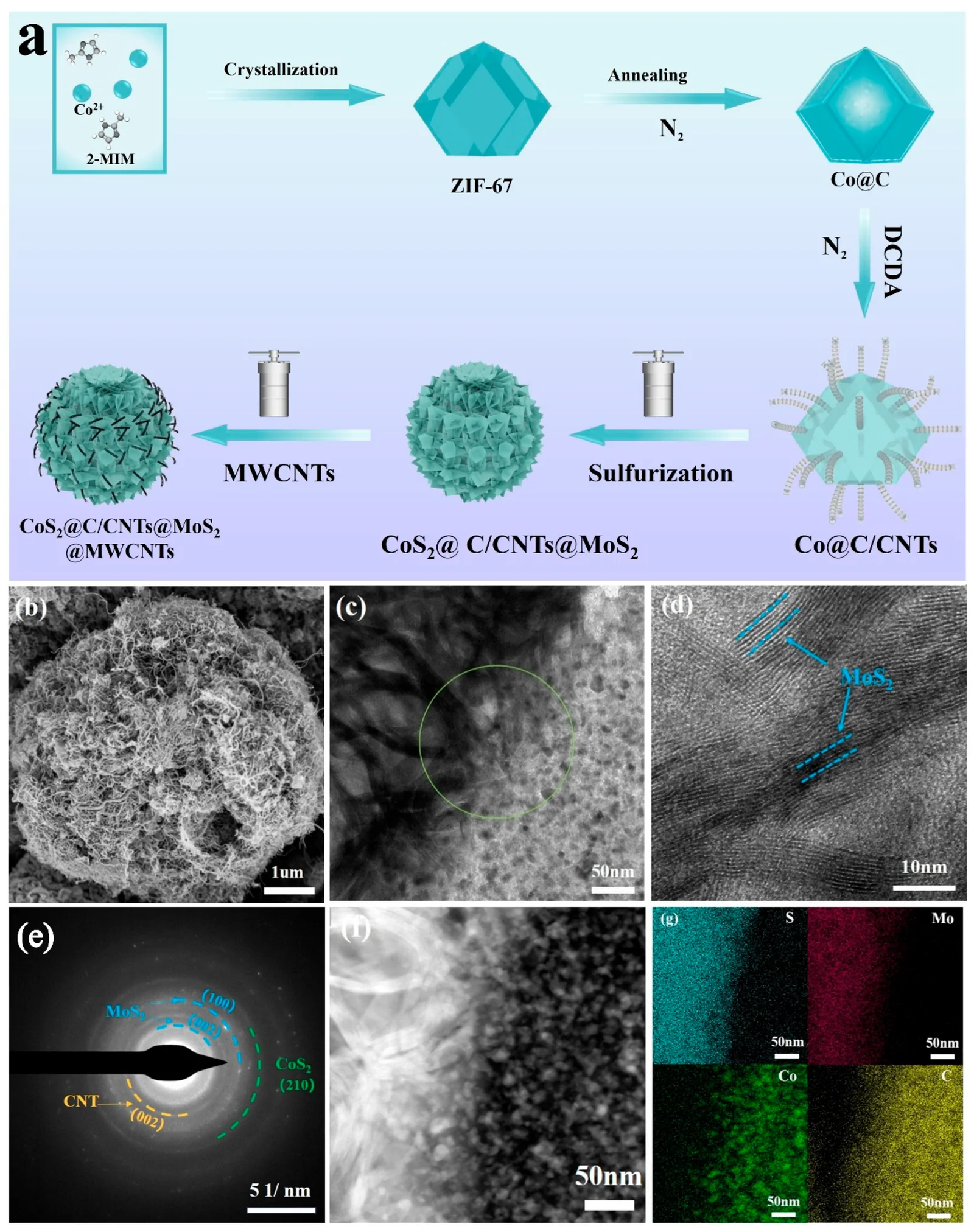
(**a**) schematic of the synthesis route and (**b**) scanning electron microscopy (SEM) image of CoS_2_@C/CNTs@MoS_2_@MWCNTs composite; (**c**) high−resolution transmission electron microscopy (TEM) image of CoS_2_@C/CNTs@MoS_2_ after focused ion beam instrument cutting; (**d**) HR−TEM image of MoS_2_; (**e**) Electron diffraction pattern (embedded SAED pattern); (**f**,**g**) EDS elemental mapping images.

The synthesis pathway of the hierarchical CoS_2_@C/CNTs@MoS_2_@MWCNTs composite is schematically illustrated in Figure 1a, highlighting the evolution from the ZIF-67 precursor to a core–shell nanoflower with an outer MWCNT network. The synthesis began with the formation of ZIF-67 polyhedra (Figure S1a,b), which were then carbonized to obtain Co@C (Figure S1c,d). Subsequently, dicyandiamide was thermally decomposed to generate nitrogen-containing reactive gaseous species, which, under the catalytic effect of the Co nanoparticles, induced the in-situ growth of N-doped carbon nanotubes, thereby converting the precursor into the Co@C/CNTs hybrid intermediate (Figure S1e,f). In this intermediate, the Co nanoparticles and CNTs constitute a highly conductive and mechanically robust internal scaffold that supports the subsequent deposition of MoS_2_. Upon hydrothermal introduction of Mo and S sources, MoS_2_ nanosheets nucleated on the CNT surfaces while the Co nanoparticles were simultaneously sulfurized to CoS_2_. This interfacial anchoring effect directed the conformal growth of MoS_2_, resulting in a well-defined CoS_2_@C/CNTs@MoS_2_ core–shell nanoflower with an average diameter of approximately 2 μm (Figure S2a,b). A fractured cross-sectional image of CoS_2_@C/CNTs@MoS_2_ (Figure S3a,b) confirms that the inner CoS_2_@C/CNTs core has a diameter of about 1.2–1.5 μm, while the MoS_2_ shell, supported by protruding CNTs, is roughly 0.1–0.2 μm thick. To build the composite architecture, a secondary layer of commercial MWCNTs was uniformly deposited onto the MoS_2_ surface via electrostatic adsorption (Figure S4). Following adsorption, the outer MWCNTs become physically entangled and interact with the underlying structure through π–π stacking and van der Waals forces resulting in a robust, cross-linked network that significantly enhances both mechanical integrity and electronic conductivity.

To investigate the microstructural characteristics of the CoS_2_@C/CNTs@MoS_2_ and CoS_2_@C/CNTs@MoS_2_@MWCNTs composites, a combination of SEM, TEM, and FIB-SEM was employed across multiple magnifications. As shown in Figure 1b, SEM images reveal a uniformly coated CNT network, which is critical to reinforce the structural robustness of MoS_2_ electrode. This coating effectively mitigates performance degradation by buffering volume expansion and preventing structural collapse during cycling. Additionally, it forms a continuous conductive framework that enhances charge carrier mobility, thus enhancing the electrochemical kinetics of MoS_2_ electrode. In addition, the tap density of spherical O_3_-type NaNi_0.4_Fe_0.2_Mn_0.4_O_2_ cathode material has been reported to be 1.187 g cm^−3^ [36], while the value obtained in this work is 0.8 g cm^−3^, which are within the same order of magnitude, indicating that the packing density of our material is reasonably competitive. This relatively high value arises from the uniform coating of MWCNTs on the dense MoS_2_ nanospheres rather than the formation of a loose three-dimensional network, which is favorable for accommodating more active material within a given electrode volume [37].

The internal configuration is further clarified in Figure 1c, where a FIB-milled cross-section of a CoS_2_@C/CNTs@MoS_2_ nanoflower distinctly exhibits a core-shell architecture. The inner core comprises CoS_2_@C and CNTs, encapsulated by an outer shell of MoS_2_ nanosheets. HR-TEM imaging in Figure 1d reveals well-defined lattice fringes with an interplanar spacing of 0.612 nm, associated with the (002) crystallographic plane of MoS_2_. Figure S5 highlights lattice spacings of 0.35 nm and 0.24 nm, which are indexed to the (002) plane of CNTs and the (210) plane of CoS_2_, respectively. The selected area electron diffraction (SAED) pattern (Figure 1e) further validates these phase assignments, displaying diffraction rings associated with CNTs (002), CoS_2_ (210), and MoS_2_ (002). Energy-dispersive X-ray spectroscopy (EDS) mapping outcomes (Figure 1f,g) demonstrate the spatial arrangement of elements, showing molybdenum along with sulfur localized in the outer regions, while carbon, cobalt, and sulfur are concentrated within the inner domain. These comprehensive structural and compositional analyses support the proposed hierarchical architecture, underscoring the synergistic reinforcement effect provided by both in situ-grown CNTs and externally integrated commercial MWCNTs.

**Figure 2:**
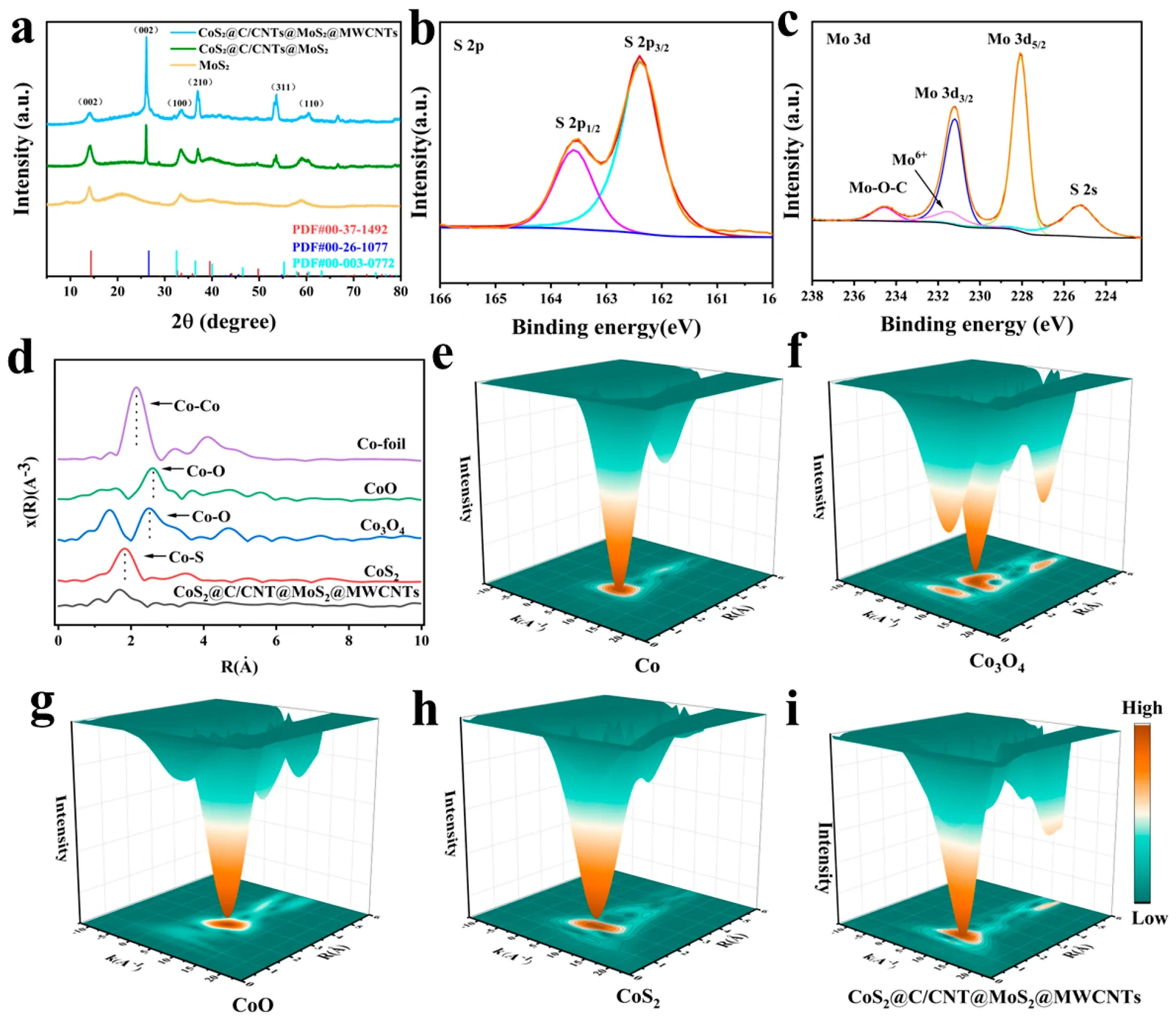
(**a**) XRD patterns of MoS_2_, CoS_2_@C/CNTs@MoS_2_, and CoS_2_@C/CNTs@MoS_2_@MWCNTs, (**b**) S 2p, (**c**) Mo 3d, (**d**) FT−EXAFS spectra, (**e**–**i**) Wavelet transform of the k3−weighted EXAFS data of Co, CoO, Co_3_O_4_, CoS_2_, and CoS_2_@C/CNTs@MoS_2_@MWCNTs.

To verify the phase composition of the synthesized electrode materials, XRD analysis was carried out. As illustrated in Figure 2a, a prominent diffraction peak appears near 14.5°, corresponding to the (002) plane of the 2H-MoS_2_ phase (JCPDS No. 037-1472). Upon integration of CoS_2_@C/CNTs and subsequent surface modification with MWCNTs, additional diffraction peaks emerge at 26.1° and 36.87°, which results from the (002) plane of CNTs (JCPDS No. 26-1077) and the (210) plane of CoS_2_ (JCPDS No. 003-0772), respectively. These observations collectively confirm the successful formation of the CoS_2_@C/CNTs@MoS_2_@MWCNTs composite structure. To elucidate the chemical constituents and bonding states of the electrode material, XPS was carried out. The wide-scan XPS spectrum (Figure S6) reveals the existence of cobalt, molybdenum, sulfur, oxygen, nitrogen, and carbon elements. In the high-resolution N 1s spectrum (Figure S7), characteristic peaks can be observed at 398.7 and 401.2eV, corresponding to pyridinic N and pyrrolic N, respectively. This directly proves the successful incorporation of nitrogen into the carbon framework. Meanwhile, in the high-resolution C 1s spectrum (Figure S8), besides the dominant graphitic carbon (C–C) peak at 284.8 eV and the carboxyl carbon (C=O) peak at 289.1 eV, a distinct peak is present at 285.8 eV, which can be assigned to carbon bonded to nitrogen (C–N). The combined analysis of the C 1s and N 1s spectra indicates that the nitrogen originating from the ZIF-67 precursor has been effectively incorporated into the carbon matrix in multiple chemical forms after pyrolysis [35]. The incorporation of these nitrogen configurations introduces defect sites that can enhance sodium-ion adsorption and charge transfer, thereby improving the composite’s electrochemical activity [38,39]. The S 2p spectrum (Figure 2b) reveals two fitted peaks at 161.7 and 163.3 eV, associated with the S 2p_3/2_ and S 2p_1/2_ states, consistent with the presence of MoS_2_. Similarly, the Mo 3d spectrum (Figure 2c) exhibits four peaks. The doublet at 228.1 eV (Mo 3d_5/2_) and 231.3 eV (Mo 3d_3/2_) is assigned to Mo^4+^ of MoS_2_. The second doublet, with Mo 3d_5/2_ at 231.6 eV and Mo 3d_3/2_ at 234.5 eV, corresponds to Mo^6+^ species. The appearance of Mo^6+^ suggests partial surface oxidation of Mo^4+^, likely leading to the formation of Mo–O–C interfacial bonds between MoS_2_ and the carbon matrix [40]. The high-resolution Co 2p XPS spectrum (Figure S9) reveals two shake-up satellite (“Sat.”) peaks located at 785.6 and 801.4 eV, along with distinct spin-orbit doublets. The Co 2p_3/2_ peaks are observed at 776.6, 778.3, and 780.2 eV, and the corresponding Co 2p_1/2_ peaks at 793.3, 796.5, and 799.4 eV, respectively. Specifically, the peaks at 776.6 and 793.3 eV are attributed to Co^0^, the peaks at 778.3 and 796.5 eV to Co^2+^ species, and the peaks at 780.2 and 799.4 eV to Co^3+^ species [41]. Inductively coupled plasma optical emission spectroscopy (ICP-OES) analysis revealed that the Co content in the composite is approximately 1.24 wt%, corresponding to a CoS_2_ loading of ~2.6 wt% (Table S1).

Furthermore, to investigate the electronic configuration and bonding environment of cobalt in the composite, a combination of X-ray absorption near-edge structure (XANES) and extended X-ray absorption fine structure (EXAFS) spectroscopy was employed. Reference spectra were collected from CoS_2_, CoO, Co_3_O_4_, and metallic Co foil for comparison. In Figure S10, the Co K-edge XANES spectrum of CoS_2_@C/CNTs@MoS_2_@MWCNTs reveals an absorption edge position situated between those of metallic Co (0) and Co^2+^, suggesting a valence state for cobalt between 0 and +2. The Fourier-transformed EXAFS (FT-EXAFS) spectrum (Figure 2d) displays a prominent peak at approximately 1.7 Å, corresponding to Co–S scattering. Notably, no significant Co–Co or Co–O coordination signals were detected, confirming the predominant formation of CoS_2_ in the composite—consistent with Co 2p XPS results. Further quantitative fitting of the FT−EXAFS data (Figure S11) shows a dominant Co–S bonding shell containing ~5 neighboring atoms and a bond length of 2.22 Å, indicative of a sulfur-rich local environment typical of CoS_2_. Additionally, wavelet transform EXAFS (WT-EXAFS) contour maps (Figure 2e–i) exhibit patterns closely resembling those of pristine CoS_2_, reaffirming the consistent local coordination structure of cobalt within the composite. The presence of finely dispersed CoS_2_ nanoparticles not only contributes to the structural integrity of the electrode but also enhances sodium-ion reaction kinetics, as previously demonstrated in related studies. Complementary to the structural analysis, nitrogen adsorption-desorption isotherms (Figure S12a) reveal a significantly increased specific surface area for the CoS_2_@C/CNTs@MoS_2_@MWCNTs composite (152.56 m^2^ g^−1^), compared with that of CoS_2_@C/CNTs@MoS_2_ (37.60 m^2^ g^−1^) and pure MoS_2_ (21.54 m^2^ g^−1^). Furthermore, the pore size distribution curve (Figure S12b) indicates a higher density of mesopores with a narrower distribution profile in the fully assembled composite. This combination of high surface area and well-defined mesoporosity facilitates enhanced electrolyte penetration and ion transport, while also buffering the volumetric changes during cycling [42,43].

**Figure 3:**
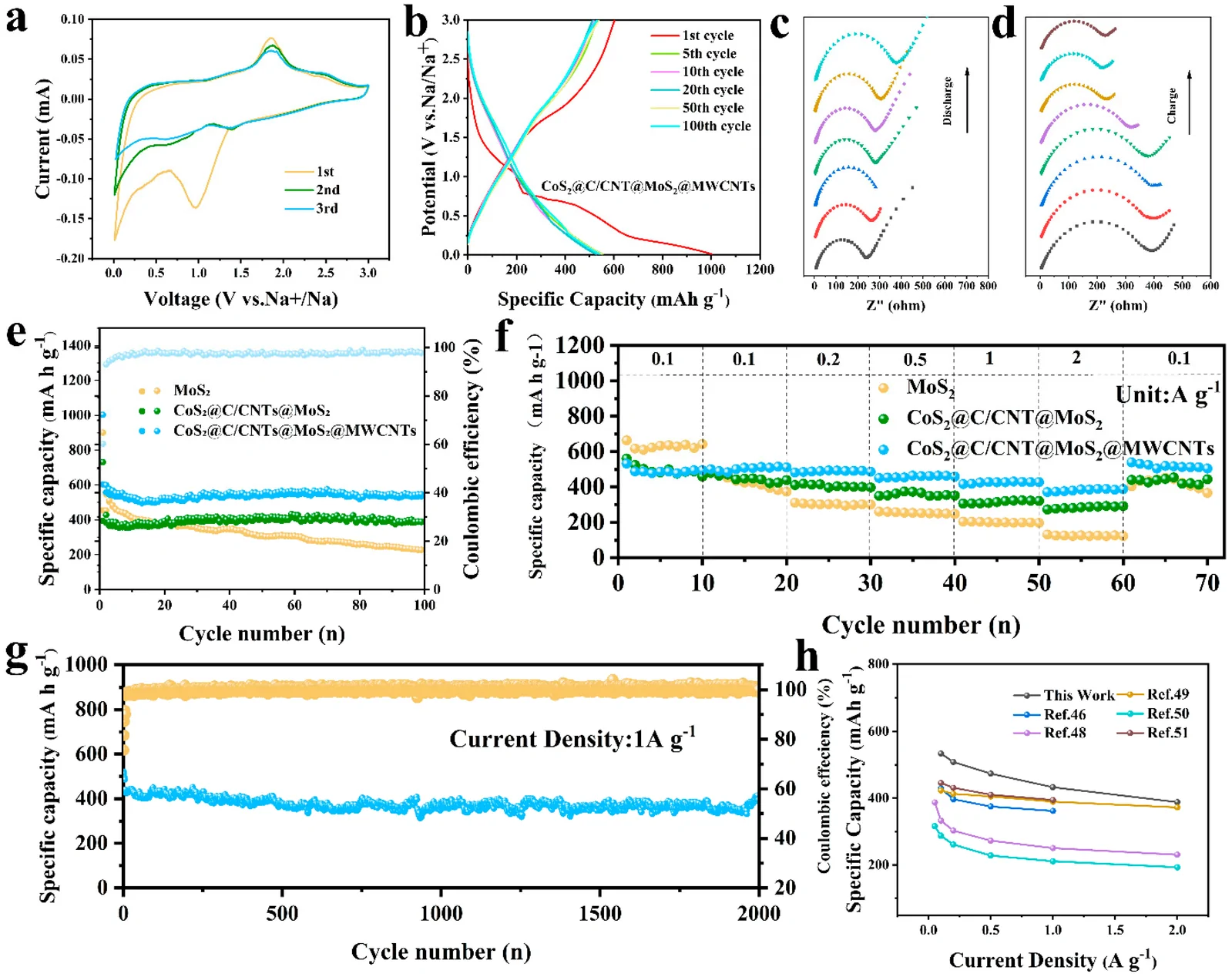
(**a**) CV profiles of CoS_2_@C/CNTs@MoS_2_@MWCNTs electrode during the first three scans; (**b**) GCD curve of the CoS_2_@C/CNTs@MoS_2_@MWCNTs electrode at 0.1 A g^−1^ (**c**) In situ EIS at discharge state and (**d**) in situ EIS at charge state, both acquired using a three−electrode cell with a Na reference electrode. (**e**) Cycling performance at 0.1 A^−1^ (**f**) Rate performance of MoS_2_, CoS_2_@C/CNTs@MoS_2_, and CoS_2_@C/CNTs@MoS_2_@MWCNTs electrodes under varying current densities. (**g**) Cycling stability at 1 A g^−1^ (**h**) Rate performance comparison of CoS_2_@C/CNTs@MoS_2_@MWCNTs electrode with reported electrodes.

The electrochemical behavior of MoS_2_, CoS_2_@C/CNTs@MoS_2_, and CoS_2_@C/CNTs@MoS_2_@MWCNTs anodes toward sodium-ion storage was systematically evaluated. Figure 3a displays the CV spectra of CoS_2_@C/CNTs@MoS_2_@MWCNTs composite, measured at a scan rate of 0.1 mV s^−1^ over cycles 1 to 3. At the beginning of cathodic sweep, a pronounced cathodic response at ~1.0 V is observed, corresponding to the intercalation of sodium ion into MoS_2_ layers to form Na_x_MoS_2_, in conjunction with a SEI layer formation [44,45].

A more pronounced cathodic peak near 0.2 V results from the subsequent conversion of Na_x_MoS_2_ into metallic Mo dispersed in a Na_2_S matrix. During the anodic sweep, a sharp oxidation peak appears at approximately 1.85 V, which is ascribed to the oxidation of NaS_x_ species. Simultaneously, the oxidation of Na_2_S to amorphous sulfur also contributes in this potential region [46]. These CV features confirm the typical multistep conversion reaction mechanism of MoS_2_ in SIBs, which are expressed below [43]:

MoS_2_ + xNa^+^ + xe^−^ ↔ Na_x_MoS_2_ (1)

Na_x_MoS_2_ + (4 − x)Na^+^ + (4 − x)e^−^ ↔ 2Na_2_S + Mo (2)

In the subsequent CV cycles, the observed decrease in current intensity is primarily attributed to irreversible capacity loss, mainly caused by the formation of the SEI and the consumption of sodium ions during initial reactions. Notably, the significant overlap between the second and third cycles demonstrates the excellent electrochemical reversibility and kinetic stability of the CoS_2_@C/CNTs@MoS_2_@MWCNTs electrode. Figure 3b shows the GCD curves of the composite electrode across different cycles (1st, 5th, 10th, 20th, 50th, and 100th) under a current density of 0.1 mA g^−1^. With the exception of the initial cycle, the discharge and charge curves exhibit a nearly complete overlap across all subsequent cycles, confirming the outstanding morphological and long-term electrochemical stability of the electrode under prolonged Na^+^ insertion/extraction cycles [47]. Based on the charge/discharge curves, the energy efficiency of this composite is about 72%. The clear voltage hysteresis is intrinsic to MoS_2_-based conversion-type anodes and persists even after adding carbon nanotubes. This result is in good agreement with the typical energy efficiencies reported for such anodes [37].

In terms of Coulombic efficiency, the CoS_2_@C/CNTs@MoS_2_@MWCNTs anode delivers an initial Coulombic efficiency (ICE) of 60.2%, which progressively increases and stabilizes near 100% in the following cycles. The lower ICE in the 1st cycle primarily results from non-reversible reactions and SEI layer development [48]. For comparison, the ICEs of pure MoS_2_ and CoS_2_@C/CNTs@MoS_2_ are notably lower, measured at 50.3% and 53.8%, respectively, highlighting the efficiency of the dual-reinforced composite structure.

To eliminate the contribution from the metallic sodium counter electrode, all EIS measurements were conducted using a three-electrode cell configuration with a sodium reference electrode. Figure 3c,d display the in-situ EIS results of the CoS_2_@C/CNTs@MoS_2_@MWCNTs electrode, capturing the dynamic evolution of interfacial resistance and ion diffusion behavior during the charge-discharge cycle of a SIB. During the discharge process, the continuous insertion of sodium ions and associated phase transitions result in a gradual increase in charge transfer resistance (Rct), reflecting the accumulation of reaction intermediates and structural rearrangements. In contrast, during charging, as sodium ions are extracted, the material volume shrinks. The flexible carbon nanotube network then helps to partially rebuild conductive pathways, which lowers the charge transfer resistance [49]. This reversible modulation of interfacial resistance highlights the favorable structural adaptability and interfacial stability of the composite electrode throughout cycling [50,51]. These observations collectively confirm the electrochemical reversibility and long-term cycling durability of the dual-reinforced CoS_2_@C/CNTs@MoS_2_@MWCNTs architecture. Moreover, in Figure S13, the Nyquist plots of the three electrodes exhibit a semicircle in the high-frequency region (attributed to charge-transfer resistance, Rct) and a straight line in the low-frequency region (associated with Warburg impedance, Zw). Fitting with an equivalent circuit yields a Rct value of 24.6 Ω for CoS_2_@C/CNTs@MoS_2_@MWCNTs, which is significantly lower than that of CoS_2_@C/CNTs@MoS_2_ and pure MoS_2_. These results suggest that the core-shell structure and the incorporation of carbon nanotubes markedly promote charge-transfer dynamics.

Figure 3e compares the cycling performance of the MoS_2_, CoS_2_@C/CNTs@MoS_2_, and CoS_2_@C/CNTs@MoS_2_@MWCNTs electrodes at a current density of 0.1 A g^−1^. During the initial 10 cycles, the CoS_2_@C/CNTs@MoS_2_@MWCNTs electrode underwent electrochemical activation, exhibiting first-cycle discharge and charge capacities of 1003.5 mA h g^−1^ and 603.5 mA h g^−1^, respectively. The relatively low ICE (60.1%) is ascribed to the large specific surface interface, porous architecture, and abundant defect sites, which promote the development of a SEI and non-reversible sodium-ion trapping—leading to substantial initial capacity loss [52]. After 100 cycles, the pristine MoS_2_ electrode exhibited pronounced capacity degradation, retaining only 223.8 mA h g^−1^. In contrast, the CoS_2_@C/CNTs@MoS_2_@MWCNTs electrode sustained a reversible capacity exceeding 500 mA h g^−1^, clearly demonstrating its structural stability and long-term durability. To clarify the role of CoS_2_, the cycling performance of three samples (pristine MWCNTs, Co@C/CNTs, and MoS_2_@MWCNTs) were compared, as shown in Figure S14. Pristine MWCNTs delivered an initial capacity of ~400 mA h g^−1^ but suffered from pronounced capacity fading. The Co@C/CNTs core maintained a stable reversible capacity of approximately 300 mA h g^−1^. In contrast, the MoS_2_@MWCNTs sample exhibited an initial capacity of ~417.5 mA h g^−1^ that rapidly faded to 216.7 mA h g^−1^. This pronounced capacity decay, compared with the stable performance of the CoS_2_-containing composite, confirms that CoS_2_ primarily acts as a structural scaffold rather than as an electrochemically active component. Within the fully integrated composite architecture, the Co@C/CNTs component functions primarily as a conductive framework and a reliable source of baseline capacity, whereas the outer MWCNT network facilitates long-range electronic percolation and provides mechanical confinement to mitigate active material detachment [53]. Consequently, the electrochemical performance of the resultant composite significantly exceeds that of the individual constituents or their physical mixture. Figure 3f further highlights the rate performance of the CoS_2_@C/CNTs@MoS_2_@MWCNTs electrode. Across a wide current density range (0.1~2.0 A g^−1^), it delivered enhanced discharge performance of 533.5~307.1 mA h g^−1^. Notably, at 2.0 A g^−1^, this performance significantly surpassed that of pure MoS_2_ (122.5 mA h g^−1^) and CoS_2_@C/CNTs@MoS_2_ (286.3 mA h g^−1^). Upon reverting the current density to 0.1 A g^−1^, the capacity rebounded to 538 mA h g^−1^. In contrast, the MoS_2_ electrode exhibited rapid capacity degradation and limited cycling reversibility, highlighting the critical role of the optimized dual-reinforced continuous conductive network in maintaining electrochemical performance.

The extended cycling stability of MoS_2_, CoS_2_@C/CNTs@MoS_2_, and CoS_2_@C/CNTs@MoS_2_@MWCNTs anodes was assessed at a current density of 1 A g^−1^ (Figure S15 and Figure 3g). After 2000 consecutive charge-discharge cycles, the electrode retained a reversible capacity of 395 mA h g^−1^ with a capacity retention of 89.9%, indicating remarkable structural stability and cycle durability. In contrast, the pristine MoS_2_ electrode exhibited rapid capacity degradation, stabilizing at merely approximately 70 mA h g^−1^ over the same period. This outstanding cycling performance can be ascribed to the unique cross-linked architecture of the composite. As shown in Figure S16, the SEM images after 2000 cycles reveal that the CNT structure remains clearly observable and intact, without any structural collapse. The inner CNTs effectively suppress MoS_2_ nanosheet agglomeration and accommodate volume expansion, while the outer MWCNTs form a robust coating that maintains the structural cohesion of CoS_2_@C/CNTs@MoS_2_ units throughout cycling. This synergistic dual-reinforcement design ensures both mechanical resilience and sustained electrochemical activity. As summarized in Figure 3h and Table S2, the CoS_2_@C/CNTs@MoS_2_@MWCNTs electrode exhibits superior sodium storage performance compared to most previously reported MoS_2_-based anodes, particularly under high current density (1 A g^−1^). These results further confirm its outstanding rate capability and long-term operational viability for advanced SIB applications [54,55,56,57,58,59].

To elucidate the sodium-ion diffusion behavior in the CoS_2_@C/CNTs@MoS_2_@MWCNTs electrode, the dependency of the electrochemical response and the sweep rate was systematically analyzed, as shown in Figure S17a. Each CV outcomes reveals two well-defined redox peaks (Peak 1 and Peak 2), whose current intensities increase progressively with rising scan rates. This behavior indicates a dominant pseudocapacitive contribution to the overall charge storage process. The charge storage behavior can be quantitatively evaluated based on the power-law dependency of peak current (i) and scan rate (v), as follows [60]:

i = av^b^ (3)

log(i) = b·log(v) + log(a)(4)

i = k_1_v + k_2_v^1/2^ (5)

The b-value approaching 0.5 or 1 reflects distinct charge storage mechanisms, corresponding to diffusion-controlled intercalation and surface-dominated capacitive behavior, respectively. In this context, k_1_v is the capacitive contribution and k_2_v^1/2^ is the intercalation-driven process. In Figure S17b, the calculated b-values for Peaks 1 and 2 are 0.83 and 0.92, respectively, suggesting that charge storage in the CoS_2_@C/CNTs@MoS_2_@MWCNTs electrode is predominantly governed by pseudocapacitive behavior. This implies that the rapid and reversible insertion/extraction of sodium ions plays a critical role in the overall electrochemical performance.

According to Equation (5), the capacitive contribution ratio is able to be quantitatively estimated by evaluating the values of k_1_ and k_2_. As shown in Figure S17c, a surge in sweep speed to a higher proportion of capacitive-dominated charge storage increases with scan rate, reaching 59.99%, 64.87%, 72.69%, 74.03%, 77.43%, 83.46%, and 88.85% at sweep speeds of 0.3, 0.5, 1.5, 2.0, 5.0, and 7.0 mV s^−1^, respectively. This progressively enhanced capacitive dominance stems from the complementary effect of engineered three-dimensional mesoporous carbon framework, which offers efficient ionic transport channels, the intimate interfacial integration between MoS_2_ nanosheets and the highly conductive CNT-based carbon matrix, and the accelerated sodium-ion insertion/extraction kinetics. These features collectively ensure fast surface redox reactions and reduced diffusion limitations, thereby enabling rate performance and reversibility in the CoS_2_@C/CNTs@MoS_2_@MWCNTs electrode [61].

**Figure 4:**
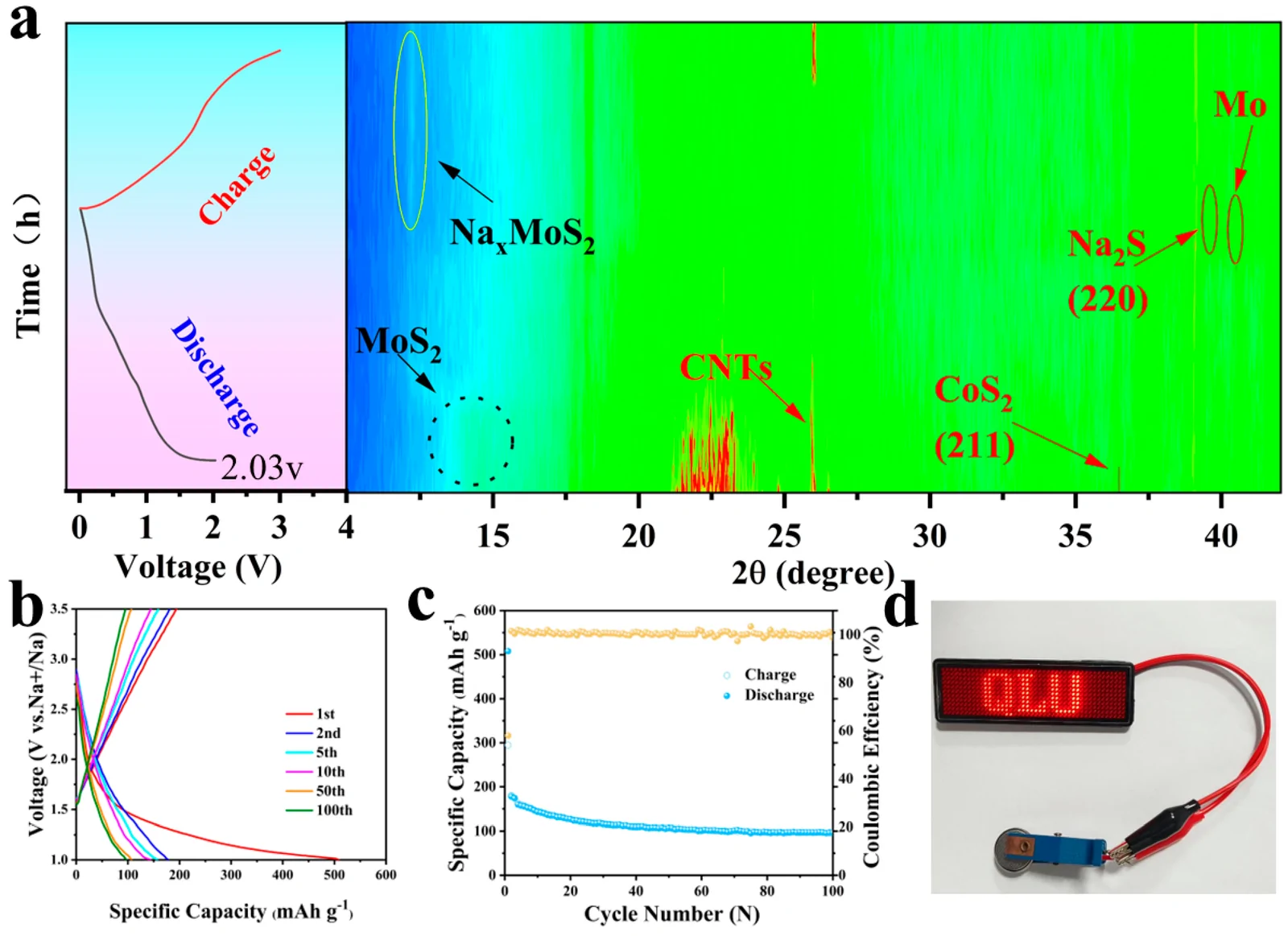
(**a**) In situ XRD evolution curves of CoS_2_@C/CNTs@MoS_2_@MWCNTs electrode during the 1st charge-discharge cycle at 0.1 A g^−1^; (**b**) GCD profiles at different cycles; (**c**) Long−term electrochemical stability of full cell at 0.2A g^−1^, (**d**) Full cell illuminating a light bulb.

To gain a deeper understanding of the sodium storage mechanism of the CoS_2_@C/CNTs@MoS_2_@MWCNTs electrode, in situ XRD measurements were conducted over a voltage window of 0.01-3.0V. Figure 4a depicts the contour plots of CoS_2_@C/CNTs@MoS_2_@MWCNTs with peak intensities color-coded with different colors/intensities to track phase evolution. In the initial discharge state, the diffraction peaks at 14.5° and 36.5° are attributed to the (002) crystal plane of MoS_2_ and the (211) crystal plane of CoS_2_, respectively. During continuous discharge, the MoS_2_ (002) peak gradually shifts toward lower angles, reflecting an increase in the interlayer spacing of its (002) crystal plane and indicating the formation of Na_x_MoS_2_ via sodium ion intercalation [40]. When discharged to 0.9 V, the MoS_2_ peak gradually weakens, while a new (110) crystal plane of metallic Mo appears at 40.5°, suggesting the onset of the conversion reaction. Upon further discharge to 0.2 V, the (220) crystal plane of Na_2_S forms at 39.6°. Notably, the disappearance of the CoS_2_ (211) peak indicates that CoS_2_ undergoes a transformation during the sodiation process. No distinct diffraction peaks for metallic cobalt are observed, which may be attributed to its low crystallinity or nanoscale dispersion. During charging, the intensities of the Na_2_S and Mo diffraction peaks gradually weaken, confirming the occurrence of a reversible conversion reaction [62]. Throughout the process, the broadening and weakening of the diffraction peaks further indicate that sodium-ion insertion induces a transition from a crystalline to an amorphous state, which is consistent with the smooth voltage plateau observed in the first-cycle discharge curve [63].

The remarkable cycling stability and favorable GCD characteristics of the CoS_2_@C/CNTs@MoS_2_@MWCNTs electrode prompted an evaluation of its applicability in a practical full battery device. To this end, a full battery was assembled via Na_3_V_2_(PO_4_)_3_(NVP) as the cathode material, as well as its electrochemical behavior was comprehensively analyzed. The operating voltage window of the NVP∥CoS_2_@C/CNTs@MoS_2_@MWCNTs full-cell was established from 1.0 to 3.5 V. The full battery maintained a stable reversible capacity of 99.8 mA h g^−1^ over 100 cycles at a current density of 0.2 A g^−1^ (Figure 4b), underscoring its stable extended cycling durability. Moreover, the charge-discharge voltage profiles of the initial cycles (Figure 4c) reveal an initial discharge capacity of 508.8 mA h g^−1^ and a corresponding charge capacity of 294.6mA h g^−1^, yielding a first-cycle Coulombic efficiency of approximately 58%. The relatively low initial performance results from the development of a SEI and non-reversible sodium loss. In Figure 4d, the fully charged full-cell was capable of powering commercial light-emitting diodes (LEDs), affirming its feasibility in practical scenarios of the CoS_2_@C/CNTs@MoS_2_@MWCNTs composite in SIB applications.

**Figure 5:**
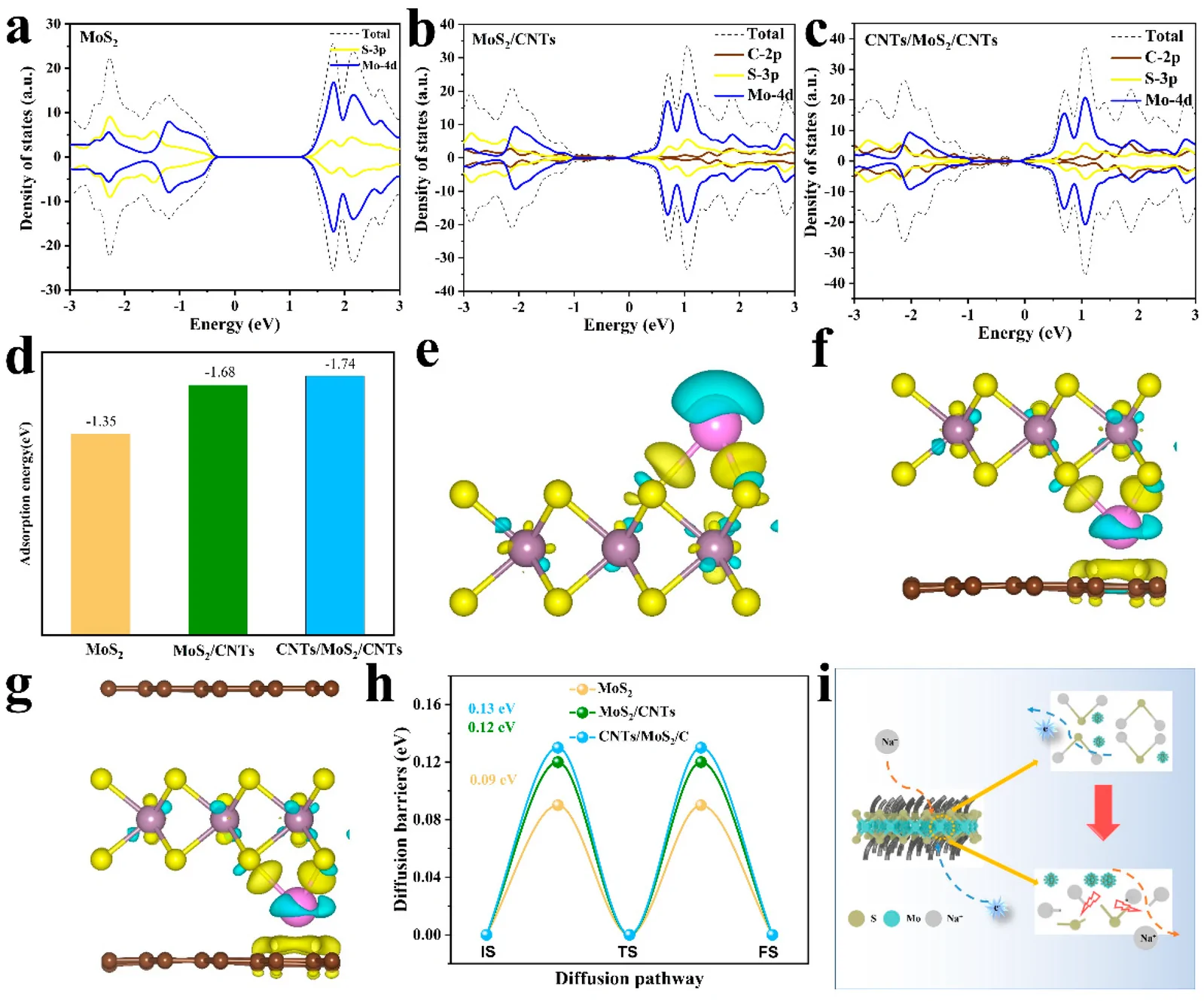
(**a**) DOS of MoS_2_ (**b**) DOS of MoS_2_/CNTs (**c**) DOS of CNTs/MoS_2_/CNTs (**d**) Sodium−ion adsorption energy (**e**–**g**) Charge density difference analysis of MoS_2_, MoS_2_/CNTs, and CNTs/MoS_2_/CNTs. (**h**) Diffusion energy barriers for MoS_2_, MoS_2_/CNTs and CNTs/MoS_2_/CNTs (**i**) Schematic illustration of the conversion reaction in CoS_2_@C/CNTs@MoS_2_@MWCNTs.

In Figure 5a–c, the density of states (DOS) calculations indicate that pristine MoS_2_ presents typical semiconducting behavior with an estimated band gap of approximately 1.52 eV, thereby restricting its intrinsic electrical conductivity. In this work, The MoS_2_ is modeled as monolayers, forming a CNTs/MoS_2_/CNTs trilayer heterostructure rather than bulk MoS_2_. In the pristine state (without Na), the equilibrium interlayer distance between MoS_2_ and C is 3.42 Å; upon insertion of a single Na atom, this distance expands to 3.85 Å due to Na intercalation. Upon the introduction of carbon-based components—particularly within the dual-carbon reinforced structure—a considerable number of carbon-derived electronic states appear within the original band gap. This electronic restructuring leads to an elevation in Fermi level, which intersects the upper energy level, signifying a transition from a semiconductor-like to a metallic electronic configuration in the CNTs/MoS_2_/CNTs heterostructure. Such a transition is critical, as the enhanced electrical conductivity fundamentally contributes to improved electrochemical kinetics, thereby offering substantial benefits for SIB anode performance.

Figure 5d presents the calculated sodium ion adsorption energies (E_a_d_s_) of pristine MoS_2_, MoS_2_/CNTs, and CNTs/MoS_2_/CNTs, aimed at evaluating the intrinsic energy storage potential of these electrode architectures. A more negative E_a_d_s_ reflects stronger sodium-ion binding affinity and greater thermodynamic favorability. The corresponding computed values are −1.35 eV for MoS_2_, −1.68 eV for MoS_2_/CNTs, and −1.74 eV for CNTs/MoS_2_/CNTs, indicating a progressive enhancement in sodium adsorption strength following the integration of conductive carbon networks. This trend aligns with the XPS results, which suggest improved chemical interaction with sodium ion in the carbon-modified composites. To further elucidate the charge redistribution associated with sodium-ion interaction, charge density difference (CDD) analysis was performed (Figure 5e–g). In these visualizations, the yellow and green regions correspond to electron accumulation and depletion, respectively. Quantitative Bader charge analysis supports these findings, yielding charge transfer values of 0.779 e for MoS_2_, 0.798 e for MoS_2_/CNTs, and 0.802 e for CNTs/MoS_2_/CNTs. The results confirm that sodium ion acts as an electron donor and that the degree of charge transfer is maximized in the dual-carbon-modified heterostructure. Collectively, these observations underscore the synergistic role of carbonaceous components in facilitating sodium-ion adsorption and improving electron transport within the composite framework.

The diffusion energy barrier of sodium ion serves as a key determinant of the rate performance in electrode systems, as it fundamentally governs the kinetics of the charge/discharge processes in SIBs. To assess this critical parameter, the climbing image nudged elastic band (CI-NEB) method was adopted, evaluating sodium-ion migration between two adjacent energetically favorable adsorption sites. The most stable adsorption site within the interlayer region between CNTs and MoS_2_ was identified as the initial state, and migration was then simulated from this site to an adjacent equivalent site as the final state (Figure S18). In Figure 5h, the computed energy barriers for sodium-ion migration in pristine MoS_2_, MoS_2_/CNTs, and CNTs/MoS_2_/CNTs reveal minimal variation, with differences of only 0.03~0.04 eV. Although the intercalation structure exhibits slightly stronger sodium-ion binding than pristine MoS_2_, leading to marginally higher barriers, the increase is not significant. This implies that while the hybrid structures enhance sodium-ion adsorption energetics—beneficial for achieving high capacity—they do not compromise ionic mobility, thereby sustaining favorable rate capability.

The underlying reaction pathway of MoS_2_ during sodiation is summarized in Figure 5i. At the initial stage of discharge, sodium ions are reversibly inserted into the van der Waals gaps of MoS_2_ layers to form Na_x_MoS_2_. This intercalation process, primarily pseudocapacitive in nature, contributes to excellent rate characteristics. As sodiation deepens, a conversion reaction takes place wherein Na_x_MoS_2_ is decomposed into metallic Mo nanoparticles embedded within a Na_2_S matrix. Upon charging, the backward transformation occurs, regenerating MoS_2_ from Mo and Na_2_S. This two-step mechanism not only contributes to a high theoretical capacity but also improves electronic conductivity through the in situ generation of metallic Mo. Meanwhile, the carbon nanotubes on both sides provide a shorter electron transmission path and buffer the electrochemical volume expansion, boosting the electrochemical performance of the electrode.

## 4. Conclusions

In summary, we successfully developed a structurally integrated CoS_2_@C/CNTs@MoS_2_@MWCNTs composite using a combination of catalytic growth and electrostatic assembly. This composite serves as a high-capacity and mechanically stable anode material for SIBs. The in-situ generated CNTs and the externally adsorbed MWCNTs are strategically arranged on opposite sides of the MoS_2_ layers, creating a dual-confinement effect that reinforces the structural framework. The embedded CNTs contribute to improved electrical conductivity, while also enhancing mechanical integrity during repeated cycling. These structural advantages provide a robust foundation for stable sodium-ion storage.

Owing to its well-engineered structural design, the CoS_2_@C/CNTs@MoS_2_@MWCNTs anode exhibits outstanding sodium storage performance, achieving a elevated specific capacity of 532.4 mA h g^−1^ following 100 cycles at 0.1 A g^−1^. Even when operated at a higher current density of 1 A g^−1^, it maintains an outstanding rate capability with a capacity of 394 mA h g^−1^, while long-term cycling tests reveal exceptional stability with sustained capacity over 2000 cycles. This remarkable combination of high capacity and long cyclic stability can be attributed to the effective buffering of volume changes and the maintained conductive network.

Insights from DFT calculations provide a mechanistic understanding of this performance. The synergistic effect of low sodium-ion diffusion barriers and strong adsorption energy in the composite facilitates efficient and rapid sodium-ion transport, even at energetically stable sites. This unique combination markedly improves rate capability. Moreover, the practical viability of this anode is demonstrated in a full-cell configuration. The CoS_2_@C/CNTs@MoS_2_@MWCNTs//NVO-C full-cell configuration demonstrates stable electrochemical performance, further underscoring the composite’s promise as a viable anode material for next-generation SIBs. The stable full-cell operation, together with the above results, confirms that the dual-confinement design effectively reconciles high capacity with long-term durability.

## Data Availability

The original contributions presented in this study are included in the article/Supplementary Materials. Further inquiries can be directed to the corresponding authors.

## Supplementary Materials

The following supporting information can be downloaded at: https://www.mdpi.com/article/10.3390/ma19173586/s1.
