# Supplementary material for: In Situ Growth CNTs and Commercialization MWCNTs Dual-Reinforced MoS2 with Cross-Link Structure for Stable Sodium-Ion Storage
Source: Materials (Basel). 2026 Aug 24;19(17):3586. doi: 10.3390/ma19173586 (PMC13566164; doi:10.3390/ma19173586)
Supplement: Supplementary file 1 [file materials-19-03586-s001.zip › materials-4462155-supplementary.pdf]

Supplementary Material

In situ growth CNTs and commercialization MWCNTs dual-reinforced MoS_2 with cross-link structure for stable sodium-ion storage

Xiao Li [a+], Nana Hu [b+], Weina Bi [a], Shilong Wen [a], Shufan Feng [a], Xuesong Zhang [a], Baogang Zhao [a], Jiaoxian Yu [a*], Jixun Xie [a*] and Jingyun Ma [a, c,*]

[a] **Shandong Provincial Key Laboratory of Chemical Energy Storage and Novel Cell Technology, School of Materials Science and Engineering, Qilu University of Technology (Shandong Academy of Sciences), Jinan 250353, PR China**

[b] **College of Chemistry and Chemical Engineering, Ocean University of China, Qingdao 266100, China**

[c] **State Key Laboratory of Crystal Material Shandong University, Jinan 250100, PR China**

[+] **These authors contributed equally to this work**

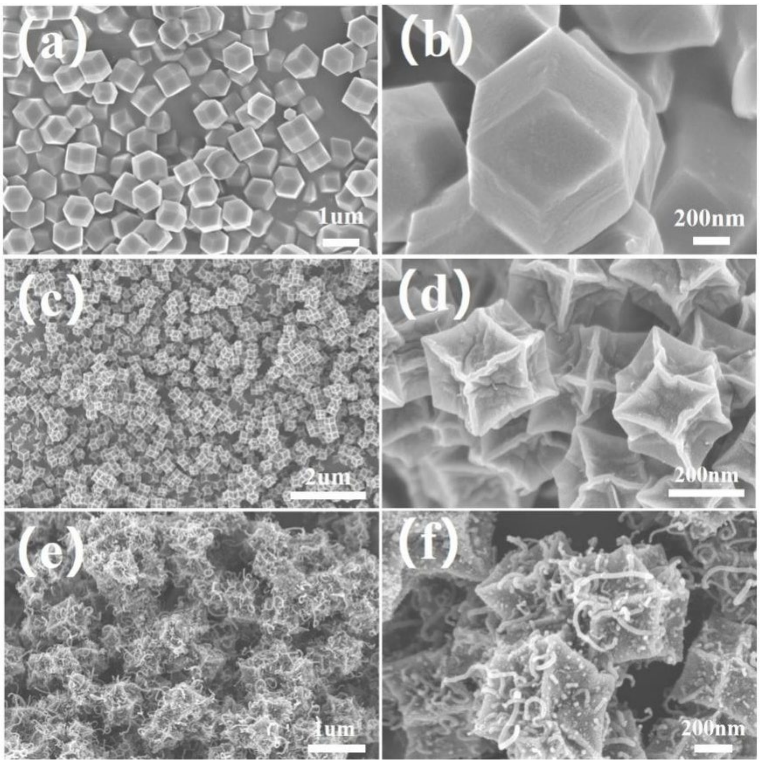

Figure S1. (a, b) SEM images of ZIF-67 at different magnifications (c, d) SEM images of ZIF-67 after calcination (e, f) SEM images of Co@C/CNTs at different magnifications.

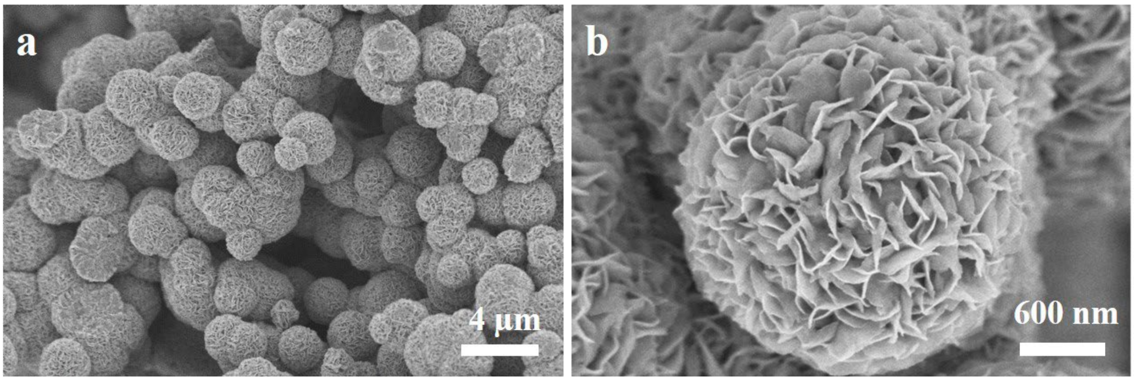

Figure S2. SEM images of CoS_2@C/CNTs@MoS_2.

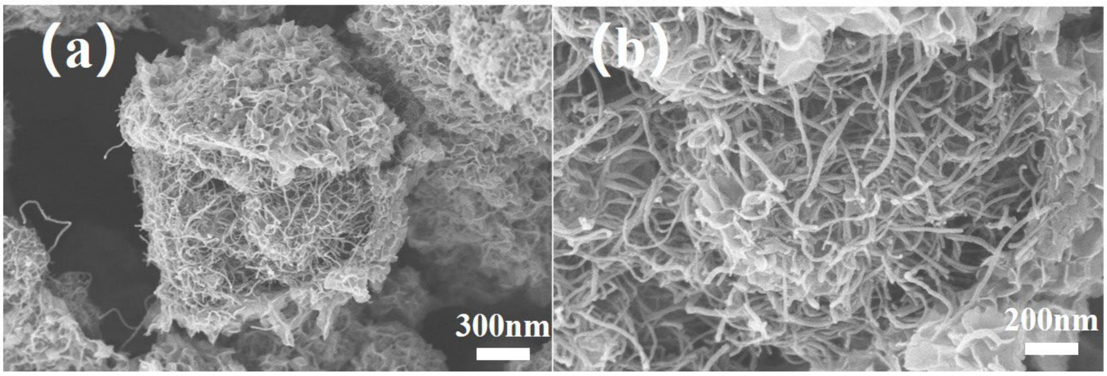

Figure S3. (a, b) SEM images of core-shell CoS_2@C/CNTs@MoS_2 nanoflowers.

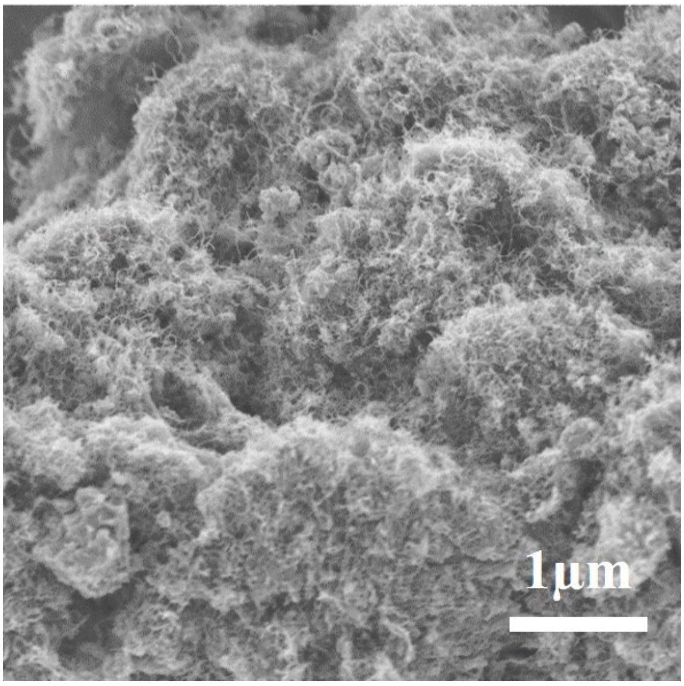

Figure S4. SEM image of CoS_2@C/CNT@MoS_2@MWCNTs composite material.

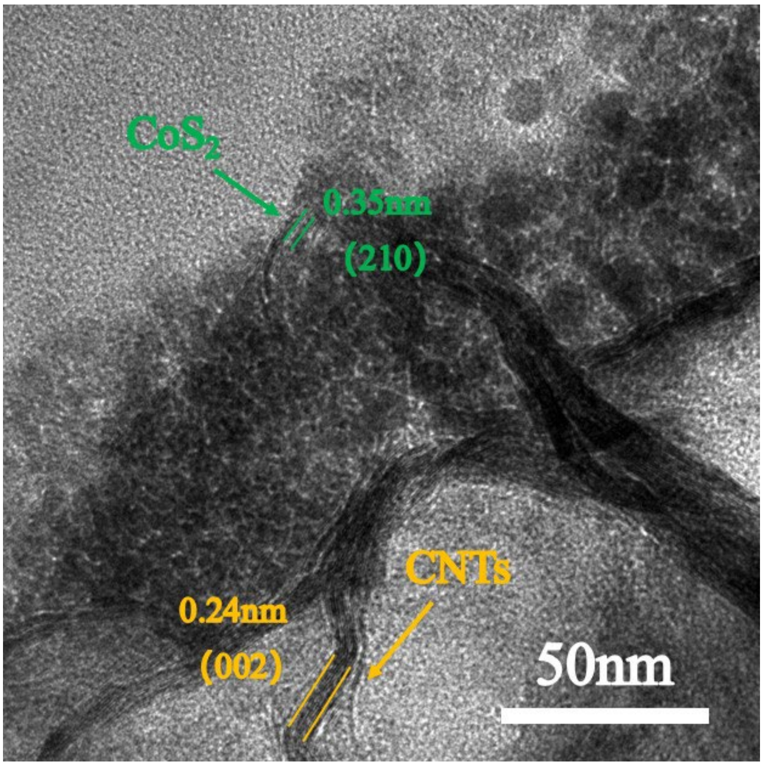

Figure S5. HR-TEM images of CoS_2@C/CNT@MoS_2.

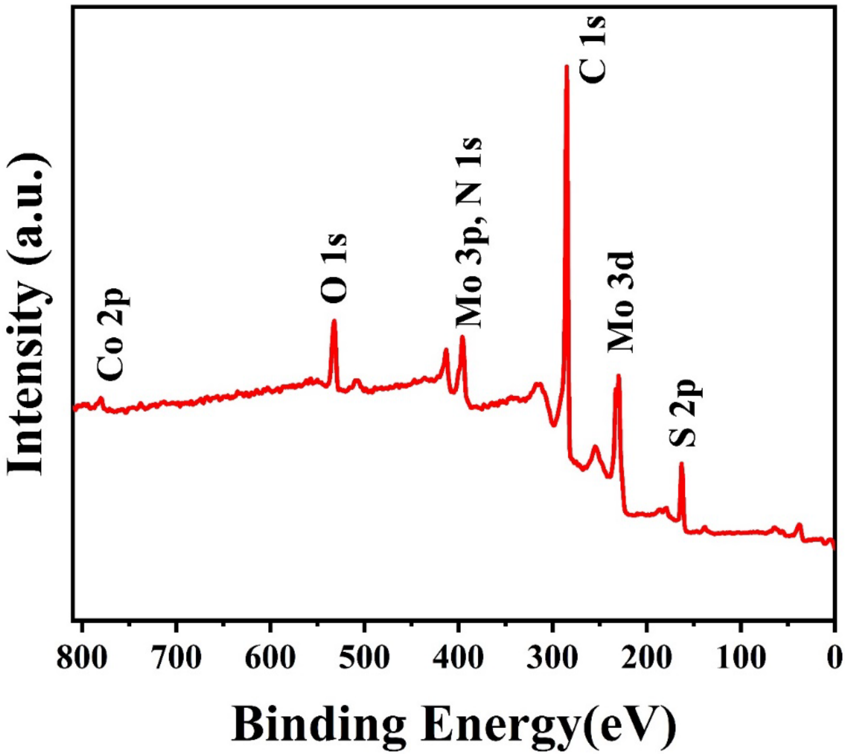

Figure S6. XPS Full Score of CoS_2@C/CNT@MoS_2@MWCNTs.

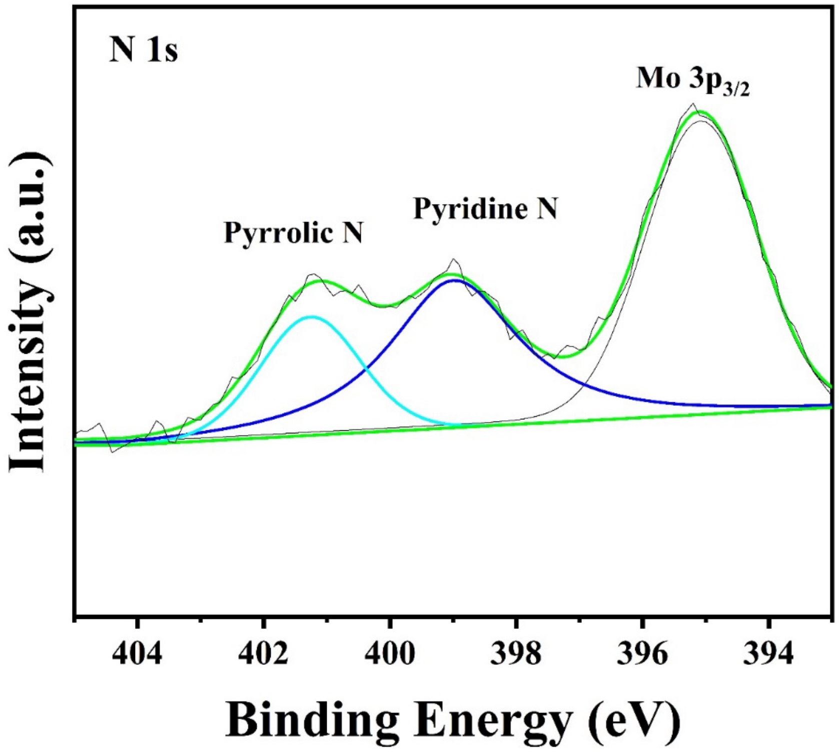

Figure S7. High-Resolution XPS Spectra of N 1s.

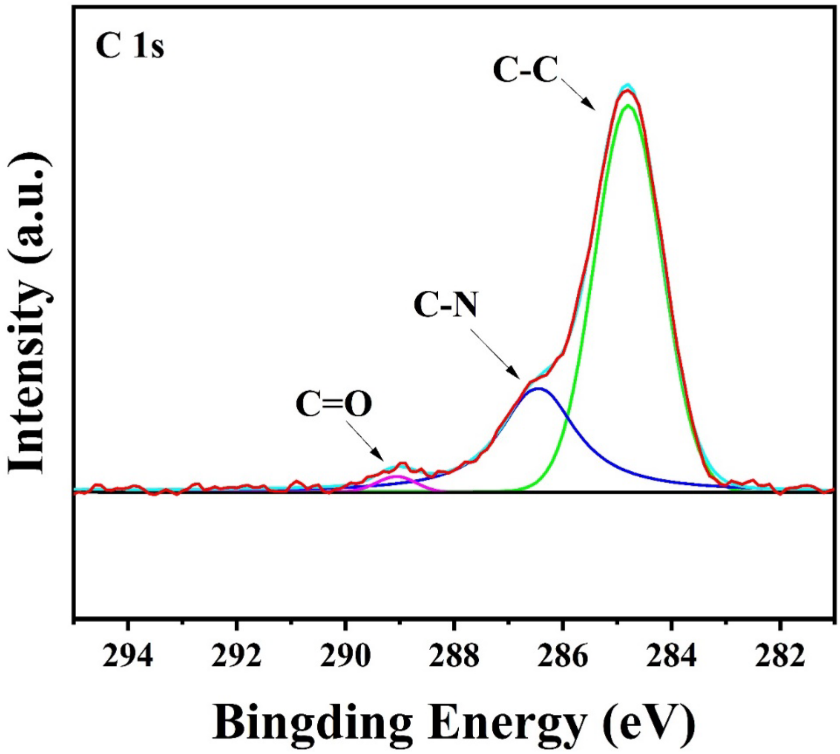

Figure S8. High-Resolution XPS Spectra of C1s.

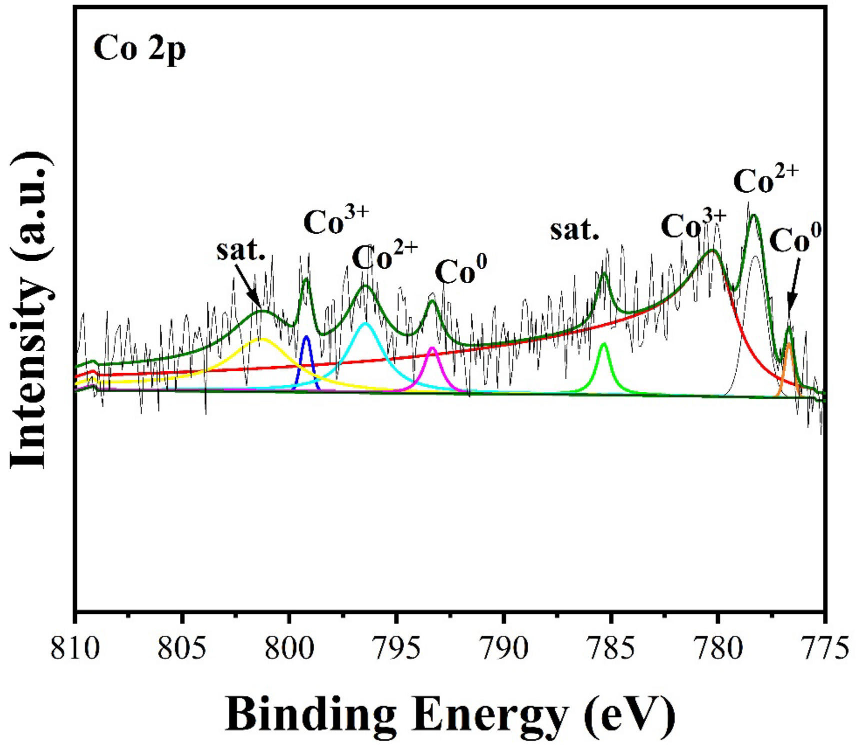

Figure S9. High-Resolution XPS Spectra of Co 2p.

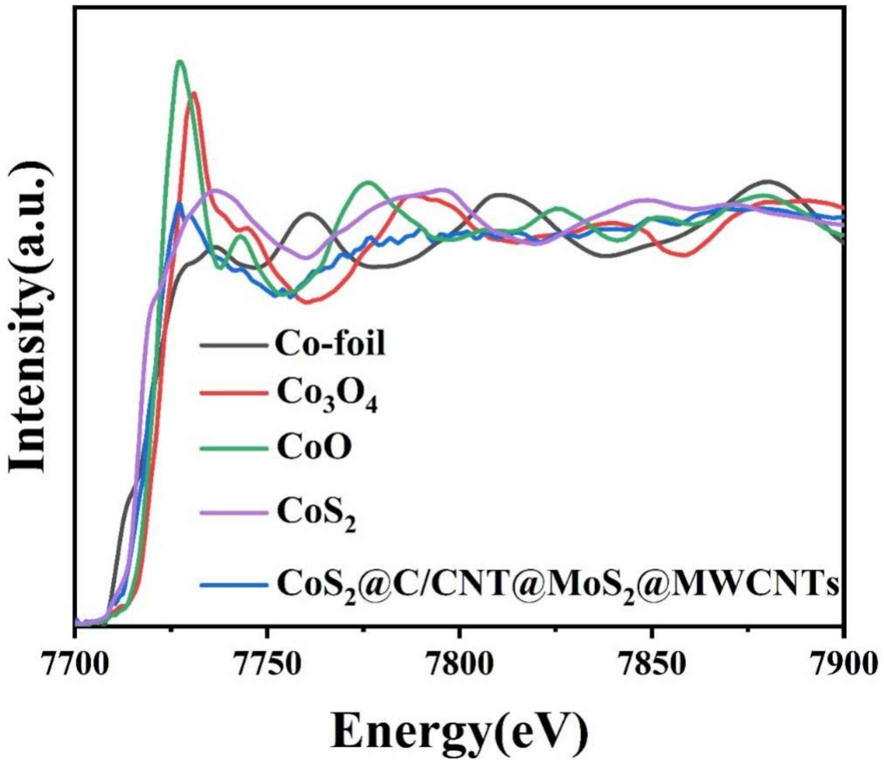

Figure S10. Co K-edge XANES profiles.

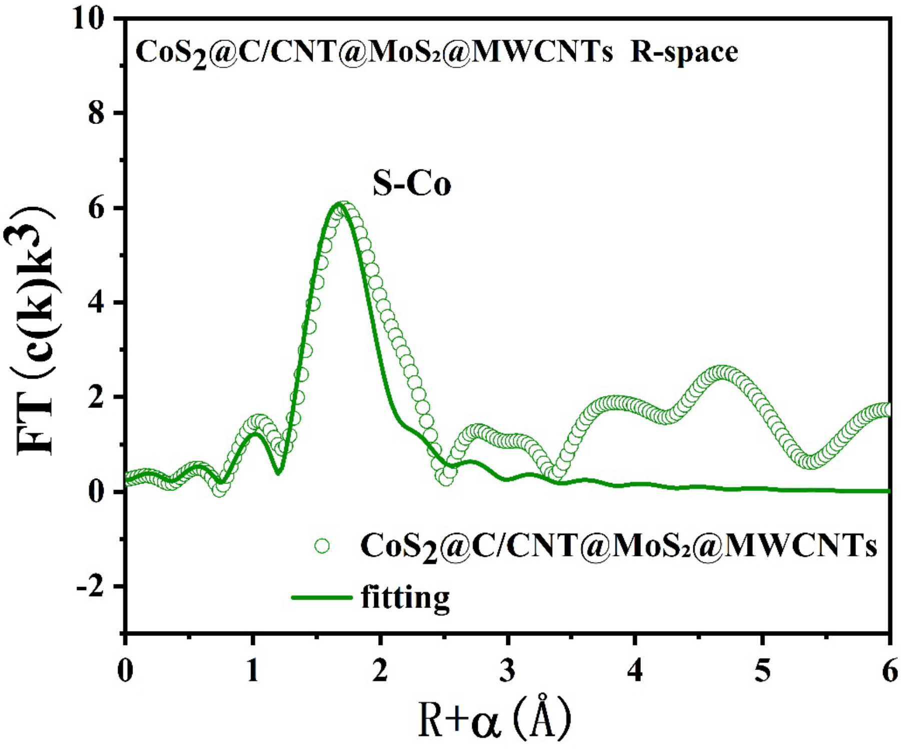

Figure S11. CoS_2@C/CNT@MoS_2@ MWCNTs EXAFS curve fitting of in R space.

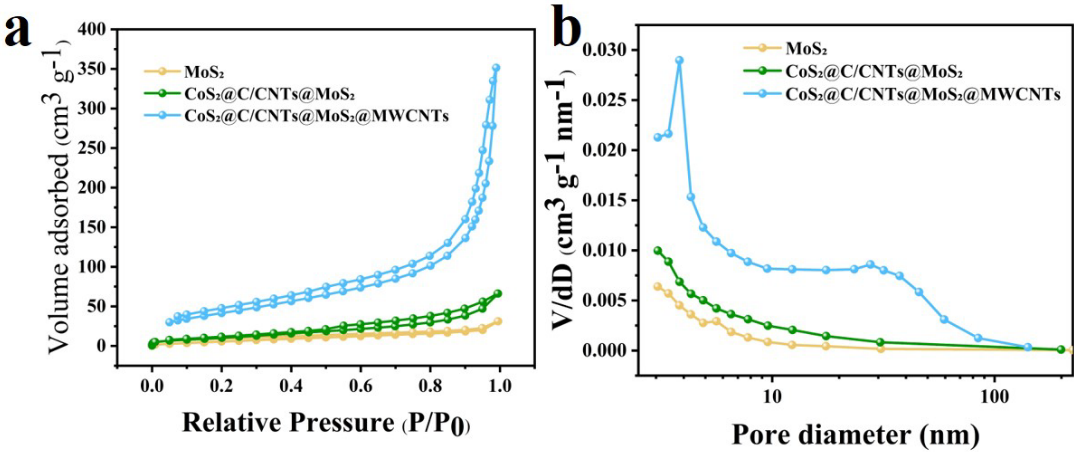

Figure S12. Nitrogen desorption isotherms of MoS_2 CoS_2@C/CNT@MoS_2 and CoS_2@C/CNT@MoS_2@MWCNTs, and (b) pore size distribution.

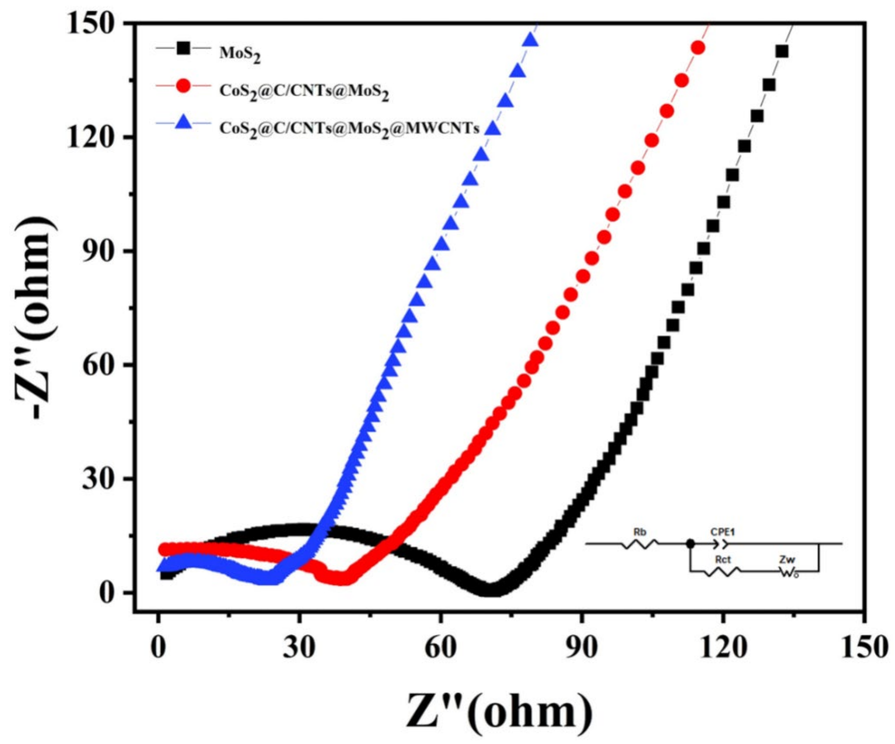

Figure S13. Electrochemical impedance spectra measured in a three-electrode cell (Na reference).

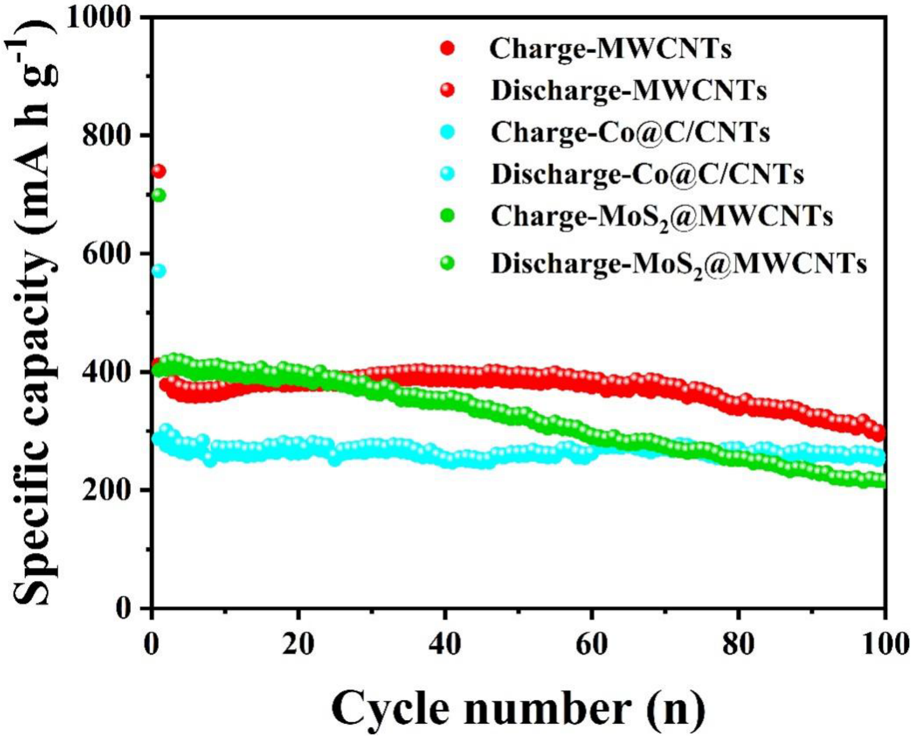

Figure S14. Cycling performance of MWCNTs, Co@C/CNTs and MoS_2@MWCNTs at 0.1 A $g^{-1.}$

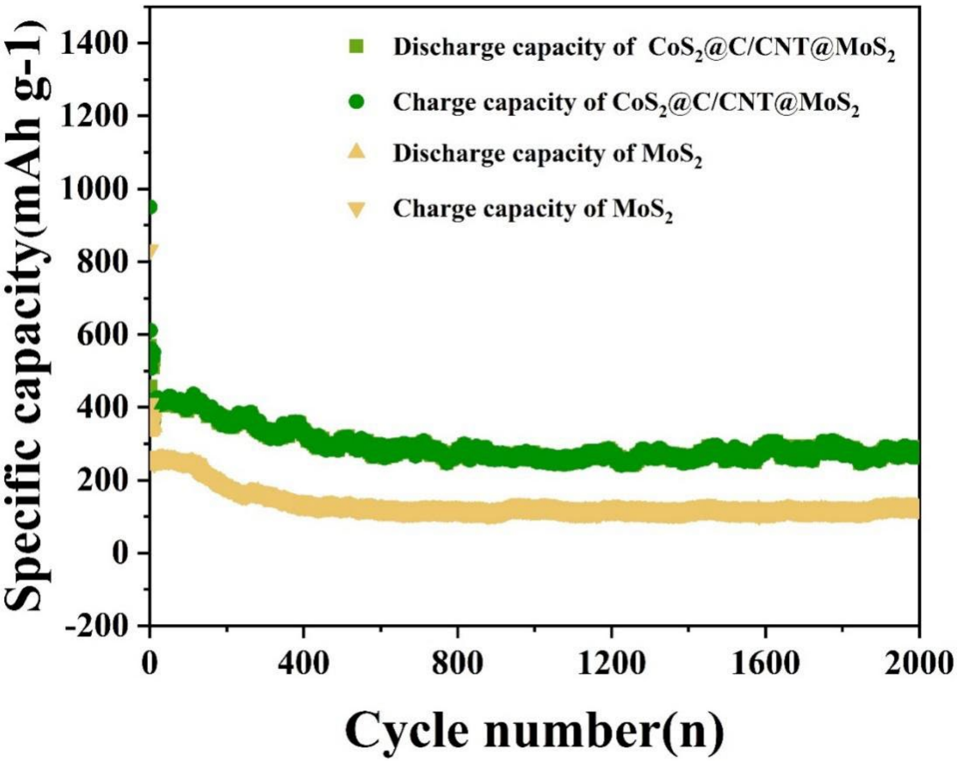

Figure S15. (a) Long cycling performance of MoS_2 and $CoS_2@C/CNT@MoS_2$ at 1 A g^{-1}.

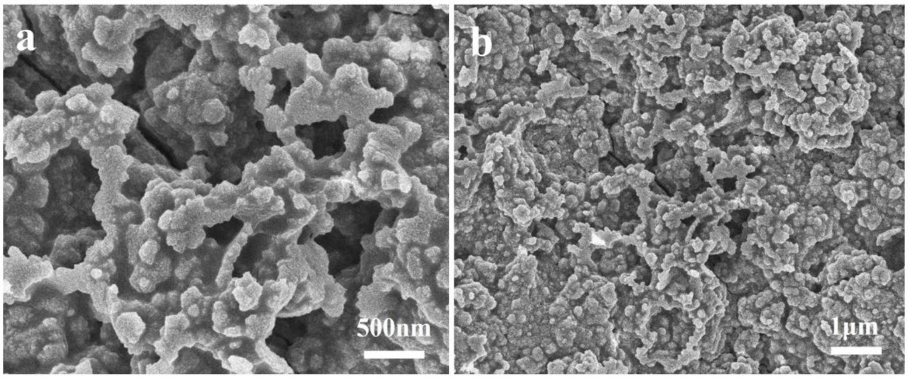

Figure S16. (a,b) SEM images of $CoS_2@C/CNT@MoS_2@MWCNTs$ electrodes after 2000 cycles at 1 A g^{-1}.

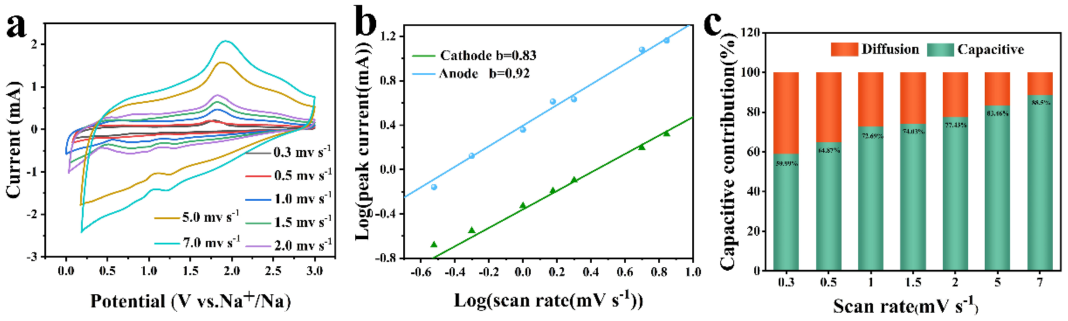

Figure S17. Electrochemical kinetic evaluation of CoS_2@C/CNTs@MoS_2@MWCNTs electrode; (a) CVs at 0.3~7.0 mV s-1; (b) Scan rate-dependent current response; (c) Pseudocapacitive contribution under varying sweep speed.

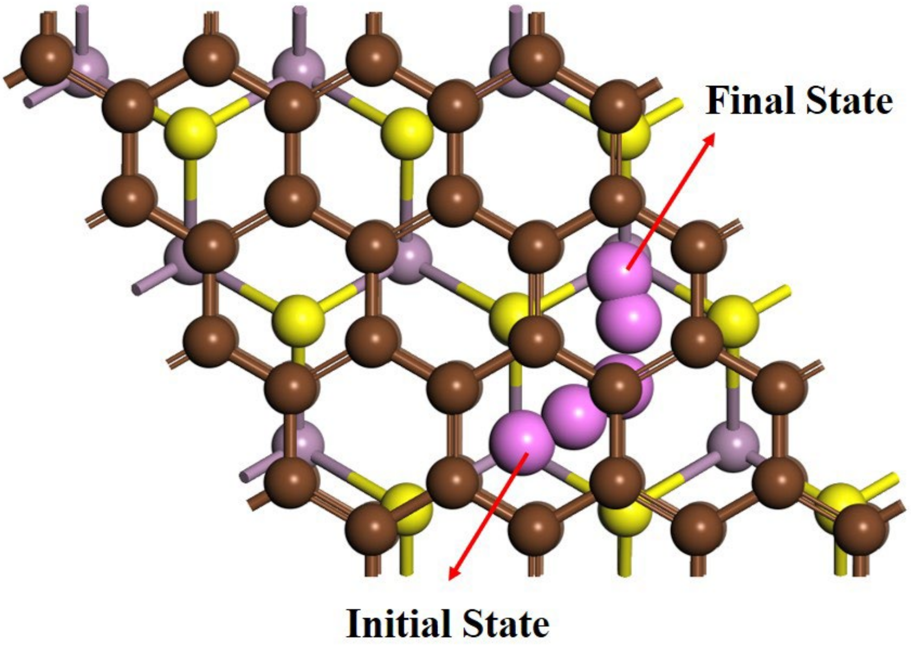

Figure S18. Na^+ diffusion pathway between the most stable adsorption site (initial state) and the adjacent equivalent site (final state) in the CNTs/MoS_2/CNTs.

Table S1. Concentrations of various elements per run, mean concentrations and relative standard deviations (RSD).

	**Co 238.892 (Aqueous Radial-IfR)**	**Mo 281.615 (Aqueous Radial-IfR)**
average concentration	2.487 ppm	27.565 ppm
concentration per run	2.497 ppm	27.872 ppm
concentration per run	2.480 ppm	27.307 ppm
concentration per run	2.485 ppm	27.516 ppm
concentration RSD	0.3%	1%

Table S2. Rate performance comparison of CoS_2@C/CNTs@MoS_2@MWCNTs electrode with reported electrodes.

	**This work**	**Ref.46**	**Ref.48**	**Ref.49**	**Ref.50**	**Ref.51**
**0.05 A g^{-1}**			387.4 mA h g^{-1}		316 mA h g^{-1}	
**0.1 A g^{-1}**	533.5 mA h g^{-1}	428.8 mA h g^{-1}	332.4 mA h g^{-1}	423.1 mA h g^{-1}	287 mA h g^{-1}	445.6 mA h g^{-1}
**0.2 A g^{-1}**	508.2 mA h g^{-1}	396.8 mA h g^{-1}	302.6 mA h g^{-1}	412.8 mA h g^{-1}	261 mA h g^{-1}	431.1 mA h g^{-1}
**0.5 A g^{-1}**	473.4 mA h g^{-1}	374.9 mA h g^{-1}	272.7 mA h g^{-1}	405.3 mA h g^{-1}	228 mA h g^{-1}	410.3 mA h g^{-1}
**1 A g^{-1}**	432.7 mA h g^{-1}	361.8 mA h g^{-1}	250.4 mA h g^{-1}	389.6 mA h g^{-1}	211 mA h g^{-1}	394.6 mA h g^{-1}
**2 A g^{-1}**	388 mA h g^{-1}		230.7 mA h g^{-1}	372.3 mA h g^{-1}	193 mA h g^{-1}	

Calculation Details

All spin-polarized density functional theory (DFT) calculations were performed by using the projector-augmented-wave (PAW) method [1] as implemented in the Vienna *ab initio* simulation package code[2]. The Perdew-Burke-Ernzerhof (PBE) functional within the generalized gradient approximation (GGA) was used to describe the exchange–correlation potential[3]. The cutoff energy of the plane wave basis set was set to 450 eV. Gaussian smearing was set for each orbital with the width of 0.1 eV. In addition, conjugate gradient algorithms were used to optimize the position of all atoms and the lattice vectors, and the convergence criterion was set to 10^{-5} eV and 0.02 eV/Å for energy and force, respectively. 4 × 4 graphene and 3 × 3 MoS_2 supercell with a vacuum thickness of ~18 Å was employed to build heterostructure models. The Brillouin zone was sampled by 3 × 3 × 1 k points (12 × 12 × 1 k-points) using the Monkhorst–Pack scheme for structural optimizations (density of states calculations). Grimme's DFT-D3 scheme [4] of dispersion correction was adopted to describe van der Waals (vdW) interactions in all systems. The climbing nudged elastic band method (CNEB) method was used for computing Na diffusion barriers[5]. The atomic structures were visualized by the VESTA[6]. The adsorption energy (E_{ads}) of Na on substrates was defined as:

$$E_{ads} = E_{tot} - E_{substrates} - E_{Na}$$

where E_{tot}, $E_{substrates}$, and E_{Na} denote the total energies of the adsorbed system, the pristine substrates, and the isolated Na atom, respectively. The more negative value means the stronger binding interaction between substrates and Na.

References

[1] P.E. Blöchl, Physical Review B 50 (1994) 17953-17979.

[2] G. Kresse, J. Furthmüller, Physical Review B 54 (1996) 11169-11186.

[3] S. Grimme, S. Ehrlich, L. Goerigk, 32 (2011) 1456-1465.

[4] S. Grimme, J. Antony, S. Ehrlich, H. Krieg, The Journal of Chemical Physics 132 (2010).

[5] G. Henkelman, B.P. Uberuaga, H. Jónsson, The Journal of Chemical Physics 113 (2000) 9901-9904.

[6] S. Maintz, V.L. Deringer, A.L. Tchougréeff, R. Dronskowski, 37 (2016) 1030-1035.
